# Overlooked *KCNQ4* variants augment the risk of hearing loss

**DOI:** 10.1038/s12276-023-00976-4

**Published:** 2023-04-03

**Authors:** Kyung Seok Oh, Jae Won Roh, Sun Young Joo, Kunhi Ryu, Jung Ah Kim, Se Jin Kim, Seung Hyun Jang, Young Ik Koh, Da Hye Kim, Hye-Youn Kim, Murim Choi, Jinsei Jung, Wan Namkung, Joo Hyun Nam, Jae Young Choi, Heon Yung Gee

**Affiliations:** 1 Department of Pharmacology, Graduate School of Medical Science, Yonsei University College of Medicine, Brain Korea 21 Project, Seoul, 03722 Republic of Korea; 2 Yonsei University College of Pharmacy, Incheon, 21983 Republic of Korea; 3 Department of Otorhinolaryngology, Yonsei University College of Medicine, Seoul, 03722 Republic of Korea; 4 Department of Biomedical Sciences, Seoul National University College of Medicine, Seoul, 03080 Republic of Korea; 5 Department of Physiology, Dongguk University College of Medicine, Gyeongju, 38066 Republic of Korea; 6 Channelopathy Research Center (CRC), Dongguk University College of Medicine, Gyeonggi-do, 10326 Republic of Korea

**Keywords:** Functional genomics, Medical genomics, Neurological disorders

## Abstract

Pathogenic variants of *KCNQ4* cause symmetrical, late-onset, progressive, high-frequency-affected hearing loss, which eventually involves all frequencies with age. To understand the contribution of *KCNQ4* variants to hearing loss, we analyzed whole-exome and genome sequencing data from patients with hearing loss and individuals whose hearing phenotypes were unknown. In *KCNQ4*, we identified seven missense variants and one deletion variant in 9 hearing loss patients and 14 missense variants in the Korean population with an unknown hearing loss phenotype. The p.R420W and p.R447W variants were found in both cohorts. To investigate the effects of these variants on KCNQ4 function, we performed whole-cell patch clamping and examined their expression levels. Except for p.G435Afs*61, all *KCNQ4* variants exhibited normal expression patterns similar to those of wild-type KCNQ4. The p.R331Q, p.R331W, p.G435Afs*61, and p.S691G variants, which were identified in patients with hearing loss, showed a potassium (K^+^) current density lower than or similar to that of p.L47P, a previously reported pathogenic variant. The p.S185W and p.R216H variants shifted the activation voltage to hyperpolarized voltages. The channel activity of the p.S185W, p.R216H, p.V672M, and p.S691G KCNQ4 proteins was rescued by the KCNQ activators retigabine or zinc pyrithione, whereas p.G435Afs*61 KCNQ4 proteins were partially rescued by sodium butyrate, a chemical chaperone. Additionally, the structure of the variants predicted using AlphaFold2 showed impaired pore configurations, as did the patch-clamp data. Our findings suggest that *KCNQ4* variants may be overlooked in hearing loss that starts in adulthood. Some of these variants are medically treatable; hence, genetic screening for *KCNQ4* is important.

## Deafness: Unrecognized mutations associated with adult-onset hearing loss

A genomic survey of the general population reveals novel damaging mutations in a gene linked to this condition, and suggests that such mutations may be surprisingly common. The *KCNQ4* gene plays a critical role in the hair cells that translate sounds into nerve signals, and defects in this gene contribute to adult-onset hearing loss. Kyung Seok Oh from Yonsei University, Seoul, South Korea, and colleagues have identified several new mutations that impair the function of *KCNQ4*. By characterizing how the resulting mutant proteins behaved in cultured cells and using computational modeling strategies the researchers found that the effects of several of these mutations might be mitigated by KCNQ activators. Some mutations were more common than expected, indicating that some cases of apparent age-related hearing loss may actually be genetic in origin.

## Introduction

Hearing loss is one of the most common sensory disorders, and more than half of congenital cases result from hereditary causes of hearing loss^1–3^. More than 125 genes (https://hereditaryhearingloss.org/) are implicated in nonsyndromic hearing loss (NSHL); approximately 30% of these genes are associated with autosomal dominant NSHL (ADNSHL)^4,5^. These genes play essential roles in maintaining normal hearing function, such as development, morphology, potassium (K^+^) recycling, and homeostasis in the inner ear^6–8^.

KCNQ4, also known as K_V_ 7.4, is a voltage-gated K^+^ channel that plays an important role in auditory function. KCNQ4 is mostly localized in the basolateral membrane of outer hair cells (OHCs) and is involved in K^+^ recycling and repolarization^9–11^. Without KCNQ4, OHCs gradually degenerate because of cellular stress caused by continuous depolarization, resulting in progressive hearing loss^12,13^.

KCNQ4 is one of the most frequently mutated genes in NSHL, resulting in autosomal dominant NSHL (DFNA2)^14^, a typically late-onset, initially high-frequency loss that progresses over time^10,15^. The KCNQ4 protein consists of six transmembrane domains, and most mutations are clustered in the pore region^16^. These mutations affect channel activity, and inhibition of the normal function of the wild-type KCNQ4 channel disrupts K^+^ recycling in the inner ear^16–18^. Therefore, as a therapeutic strategy for DFNA2 caused by *KCNQ4* mutations, potassium channel openers, including retigabine, zinc pyrithione, and MaxiPost, have been used^7,16,17^.

Trafficking deficiency and protein misfolding are the most common pathogenic mechanisms underlying the development of several diseases. Misfolded proteins, such as cystic fibrosis transmembrane conductance regulator, *CFTR* (F508del) and *SLC26A4*/pendrin (p.H723R), fail to enter the secretion pathway, which ultimately causes clinical disorders^19,20^. Mutations in both CFTR and pendrin result in protein misfolding, endoplasmic reticulum (ER) retention, and endoplasmic reticulum-associated degradation (ERAD), thereby causing loss of ion transport function. The KCNQ4 variants p.L274H, p.L281S, and p.G296S have been reported to reduce cell surface expression of KCNQ4 proteins, which is restored by overexpression of HSP90, a molecular chaperone required for the folding and trafficking of various membrane proteins^15^.

In this study, we identified seven missense *KCNQ4* mutations in a cohort of patients with hearing loss. Moreover, we hypothesized that hearing loss-causing variants of *KCNQ4* may be overlooked in individuals with unknown hearing loss owing to late onset and progressive clinical phenotypes. Thus, we analyzed a genomic database containing a Korean cohort with unknown hearing loss and identified 14 missense mutations in *KCNQ4*. We investigated the K^+^ channel activity and examined the pathogenicity of the mutations in comparison with p.L47P, a previously reported pathogenic loss-of-function KCNQ4 variant^17^. We also investigated the effects of KCNQ4 activators on the K^+^ channel activity of the pathogenic variants. Moreover, we identified one deletion mutation in the Yonsei University Hearing Loss (YUHL) cohort, which was partially rescued upon treatment with sodium butyrate. Finally, structure prediction using AlphaFold2 showed substantially different pore configurations in variants whose currents were impaired. Our data suggest that KCNQ4-mediated hearing loss could be overlooked because of the late-onset clinical phenotype, and some of the variants might be medically treatable using KCNQ4 channel openers.

## Materials and methods

### Patients and diagnosis of sensorineural hearing loss

This study was approved by the Institutional Review Board of Severance Hospital, Yonsei University Health System (IRB #4-2015-0659). We obtained informed written consent from individuals with hearing loss for participation in this study and publication of their clinical data and enrolled them in the YUHL cohort. All patients registered in the YUHL cohort had hearing loss and were referred to Severance Hospital for further evaluation and treatment. Pure-tone audiograms and speech audiograms were obtained for all patients and their affected and unaffected family members. Pure-tone air (250–8000 Hz) and bone conduction (250–4000 Hz) thresholds were measured using a clinical audiometer in a double-walled audio booth. The degree of hearing loss was determined by averaging the air conduction thresholds at 500, 1000, 2000, and 4000 Hz. The auditory, steady-state response was also determined in young babies. Temporal bone computed tomography and magnetic resonance imaging were performed to evaluate inner ear abnormalities.

### Control data collection

Whole-exome sequencing (WES) and whole-genome sequencing (WGS) data for Korean individuals were obtained from four independent reference groups. The WES reference dataset included WES data from the Clinical & Omics Data Archive (CODA) and control data from Seoul National University (SNU), comprising data for 298 and 445 individuals, respectively. Both datasets were deposited in FASTQ format and analyzed using the same WES pipeline as that used for the case group. The WGS reference dataset, including data from the Korea National Institute of Health (KNIH; *n* = 397) and WGS data from CODA (*n* = 252), were provided in the variant calling format (VCF). Only the variants in the coding region of *KCNQ4* (chr1:41249766-41304195 in hg19 and chr1:40784094-40838523 in hg38) were extracted using VCFtools (v.0.1.17) and annotated using ANNOVAR (version 2018-04-16).

### Variant calling and selection

Genomic DNA was extracted from peripheral blood obtained from the affected individuals and their parents (whenever available) using RBC lysis, cell lysis, and protein precipitation solutions (iNtRon Biotechnology, Inc., Seongnam-si, Korea). WES and variant filtering were performed using an Agilent SureSelect V5 enrichment capture kit (Agilent Technologies, Santa Clara, CA, USA) with sequencing on an Illumina HiSeq 2500 platform (151 bases, Bio-Rad). GATK best-practice pipelines were used to generate a binary alignment map (BAM) and VCF files from raw unmapped reads. The human reference genome GRCH37/hg19 was used to align the reads using the Burrows–Wheeler Aligner (BWA-MEM) algorithm. To filter out low-quality single nucleotide variants (SNVs), a filter was applied with a depth of coverage (DP) ≥ 5 and a genotype quality (GQ) ≥ 20. The variants were annotated using ANNOVAR software. To identify rare variants of unknown significance, variants were filtered using minor allele frequency (MAF) in the genome aggregation database (gnomAD) with a cutoff of 0.0005. The variants were prioritized using various in silico prediction scores (shown in Table 1). Interpretation of variants was based on the clinical interpretation of the deafness variation database. If no interpretation was registered, the identified variants were classified as variants of unknown significance, and functional studies were performed.

**Table 1:** *KCNQ4* variants detected in individuals with autosomal dominant hearing loss.

Group^a^	Individual	Sex	Age of onset (years)	Nucleotide change^b^	Amino acid change	Exon	Zygosity	Amino acid sequence conservation	dbSNP^c^	ESP^d^	gnomAD^e^	KRGDB^f^	PP2^g^	MT^h^	SIFT^i^	CADD^j^	DVD^k^
YUHL	YUHL37-21	M	Late 20 s	c.991 C > T	p.Arg331Trp	7/14	Het	*Danio rerio*	rs1178772384	ND	0	ND	Dam (1)	DC (1)	Del (0)	29.2	Unknown significance
	YUHL206-21	F	Early 50 s	c.1339 C > T	p.Arg447Trp	10/14	Het	*Gallus gallus*	rs758333323	ND	0.0000335	ND	Dam (0.446)	Neu (0.98)	Del (0.02)	24.7	Unknown significance
	YUHL261-21	F	Early 10 s	c.1304del	p.Gly435AlafsTer61	10/14	Het	*Xenopus tropicalis*	ND	ND	ND	ND	ND	ND	ND	ND	ND
	YUHL450-21	F	Early 60 s	c.1258 C > T	p.Arg420Trp	9/14	Het	*Mus musculus*	rs576041348	ND	0.0001813	0.00136364	Dam (0.548)	DC (0.9)	Del (0.02)	24.8	Unknown significance
	YUHL463-21	M	Early 30 s	c.992 G > A	p.Arg331Gln	7/14	Het	*Danio rerio*	rs1408578386	ND	0.000004102	ND	Dam (0.992)	DC (1)	Del (0)	29.6	Unknown significance
	YUHL493-21	F	Early 40 s	c.316 T > G	p.Phe106Val	1/14	Het	*Danio rerio*	rs189892658	ND	0.00007615	0.000454545	Dam (0.968)	DC (1)	Del (0)	25.3	Unknown significance
	YUHL512-21	M	Early 50 s	c.2014 G > A	p.Val672Met	14/14	Het	*Xenopus tropicalis*	rs754076761	ND	0.00007961	ND	Dam (0.736)	DC (0.9)	Del (0.04)	25.2	Pathogenic
	YUHL556-21	M	Early 50 s	c.140 T > C	p.Leu47Pro	1/14	Het	*Danio rerio*	rs1271250198	ND	ND	ND	Ben (0.003)	DC (1)	Del (0.03)	22.6	Pathogenic
	YUHL608-21	F	Late 10 s	c.140 T > C	p.Leu47Pro	1/14	Het	*Danio rerio*	rs1271250198	ND	ND	ND	Ben (0.003)	DC (1)	Del (0.03)	22.6	Pathogenic

*Ben* benign, *Dam* probably damaging, *Del* deleterious, *DC* disease-causing, *Het* heterozygous in the affected individual, *F* female, *M* male, *ND* no data or DNA not available, *Neu* neutral.

^a^Group indicates cohorts in which individuals with variants were found, comprising one case cohort (YUHL).

^b^Variants are numbered according to the human cDNA reference sequence NM_004700.4 (*KCNQ4*).

^c^dbSNP database (http://www.ncbi.nlm.nih.gov/SNP).

^d^Exome Sequencing Project (https://evs.gs.washington.edu/EVS/).

^e^Population frequency of KCNQ4 variants in gnomAD (https://gnomad.broadinstitute.org/).

^f^The Korean Reference Genome Database (http://coda.nih.go.kr/coda/KRGDB/index.jsp).

^g^PolyPhen-2 HumVar prediction score (http://genetics.bwh.harvard.edu/pph2/).

^h^MutationTaster (http://www.mutationtaster.org/).

^i^SIFT Sorting Intolerant from Tolerant (http://sift.jcvi.org/).

^j^phred-like scores (scaled C-scores) on the Combined Annotation-Dependent Depletion (http://cadd.gs.washington.edu/home/).

^k^Deafness variation database (https://deafnessvariationdatabase.org/).

### Plasmid construction and site-directed mutagenesis

Complementary DNA (cDNA) for human *KCNQ4* was purchased from OriGene Technologies (Rockville, MD, USA) and subcloned into the pENTR-D-TOPO vector (Invitrogen, Carlsbad, CA, USA). Expression vectors were constructed using LR clonase (Invitrogen) according to the manufacturer’s instructions, and a Myc- or FLAG-tag was inserted at the N-terminus. *KCNQ4* variant clones were generated through PCR-based, site-directed mutagenesis using a Quick Change II XL Site-Directed Mutagenesis Kit (Agilent Technologies).

### Cell culture and transfection

Human embryonic kidney 293 (HEK 293), HeLa, and Chinese hamster ovary (CHO) cells were cultured in Dulbecco’s modified essential and RPMI 1640 media supplemented with 10% fetal bovine serum and penicillin (50 IU/mL)/streptomycin (50 μg/mL) (Invitrogen). Cells were transfected with wild-type (WT) or mutant *KCNQ4* plasmids using Lipofectamine and PLUS reagent according to the manufacturer’s instructions. For electrophysiology experiments, HEK 293 cells were cotransfected with 0.9 μg of the human *KCNQ4* plasmids (0.1 μg of a green fluorescent protein (GFP) gene-containing expression plasmid to visualize the transfected cells). Experiments were performed within 24–36 h after transfection.

### Immunoblotting, surface biotinylation, and immunofluorescence

Experiments were performed as described previously^21^. Anti-BiP (ab21685), anti-β-actin (ab6276; Abcam, Cambridge, UK), anti-Myc (sc-40), and anti-aldolase A1 (sc-12059; Santa Cruz Biotechnology, Dallas, TX, USA) antibodies were purchased from commercial sources. Surface biotinylation was performed using 0.3 mg/mL EZ-Link Sulfo-NHS-SS-Biotin and NeutrAvidin (Thermo Fisher Scientific). Immunoblotting was performed using primary antibodies at a 1:1000 dilution, followed by the corresponding anti-isotype secondary antibodies (Santa Cruz Biotechnology) at a 1:2000 dilution. Signals were visualized using the SuperSignal West Pico Kit (Thermo Fisher Scientific). For immunofluorescence experiments, blocking buffer containing 10% donkey serum and 1% bovine serum albumin in phosphate-buffered saline was used, and the dilutions used for primary and fluorophore-tagged secondary antibodies were 1:100 and 1:2000, respectively. Confocal images were obtained using a Carl Zeiss LSM780 instrument, and ZEN software was used for image processing.

### Electrophysiology

Whole-cell patch-clamp methods were used to measure the channel activities of KCNQ4 WT or mutant-transfected HEK 293 cells at room temperature (22–25 °C). The cells were transferred to a bath, perfused at 5 mL/min and mounted on the stage of an inverted microscope (Ti-U; Nikon, Tokyo, Japan) equipped with a high-density mercury lamp light source for green fluorescence excitation. Microglass pipettes (World Precision Instruments, USA) were fabricated using a PP-830 single-stage glass microelectrode puller (Narishige, Japan) with a resistance of 2–5 MΩ. The liquid junction potential was rectified using an offset circuit prior to each recording. Currents were recorded using an Axopatch 200 B amplifier and Digidata 1440 A interface, digitized at 10 kHz, and low-pass filtered at 5 kHz using pClamp software 10.7 (Molecular Devices, USA). The whole-cell patch-clamp configuration was verified by measuring the series resistance to less than 10 MΩ, which was compensated before each recording. In the whole-cell configuration, the step pulse protocol with a holding potential of −80 mV, depolarization from −80 mV to 40 mV for 2 s at 10 mV increments, and tail pulse at either −50 mV or 0 mV for 0.5 s was used. The normalized conductance measured from the tail currents was fitted to the Boltzmann equation to measure the half-maximal activation voltage.

$$\frac{G}{{G_{{{{\mathrm{max}}}}}}} = A_2 + \frac{{A_1 - A_2}}{{1 + e^{(V_{{{\mathrm{h}}}} - V)/k}}},$$

where *G/G*_max_ is the normalized conductance calculated from the tail currents, *V* is the applied voltage, *V*_h_ is the half-maximal activation voltage (denoted as V_1/2_ in the manuscript), and *k* is the slope factor. *A*_*1*_ and *A*_*2*_ are the maximum and minimum values of the curve, respectively.

### Solutions

The whole-cell patch-clamp experiment was conducted using a basal extracellular bath solution containing 147 mM NaCl, 5 mM KCl, 10 mM HEPES, 10 mM glucose, 1.5 mM CaCl_2_, 1 mM MgCl_2_, and 10 mM sorbitol, adjusted to pH 7.4 using NaOH. The basal pipette solution contained 140 mM KCl, 10 mM HEPES, 10 mM EGTA, and 3 mM Mg-ATP, adjusted to pH 7.2 using KOH.

### Mutant-drug cycle analysis

We performed mutant-drug cycle analysis using double-mutant cycle analysis calculations^22,23^. Mutant-drug cycle analysis was used to calculate the free energy difference between the WT and mutant KCNQ4 channels. From the Boltzmann equation, the slope factor *k* was defined as follows:

$$k = \frac{{RT}}{{zF}},$$

where *R* is the ideal gas constant, *T* is the absolute temperature, *F* is Faraday’s constant, and *z* is the gating variance.

The Gibbs free energy from the closed to the open conformation was calculated using the following equation:

$$\Delta G = - zF\left( {V_{{{\mathrm{h}}}} - V_{{{{\mathrm{rev}}}}}} \right).$$

By combining the two equations given above, we derived the following equation:

$$G = - RT\frac{{V_{{{\mathrm{h}}}} - V_{{{{\mathrm{rev}}}}}}}{k}.$$

Here, *V*_rev_ is the reversal potential of the permeating ion, which for our experiments was K^+^. The reversal potential was calculated using the Goldman–Hodgkin–Katz equation.

$$V_{{{{\mathrm{rev}}}}} = \frac{{RT}}{F}{{{\mathrm{ln}}}}\left( {\frac{{\left[ {{{{\mathrm{K}}}}^ + } \right]_{{{{\mathrm{out}}}}}}}{{\left[ {{{{\mathrm{K}}}}^ + } \right]_{{{{\mathrm{in}}}}}}}} \right)$$

We assumed that every closed conformation had the same energy level; thus, the delta sign was deleted for ease of understanding.

The coupling energy was calculated as follows:

$$\begin{array}{l}\Delta \Delta G_{{{{\mathrm{coupling}}}}} = \Delta G_{{{{\mathrm{WT}}}} \to {{{\mathrm{WT}}}},{{{\mathrm{Drug}}}}} - \Delta G_{{{{\mathrm{Mut}}}} \to {{{\mathrm{Mut}}}},{{{\mathrm{Drug}}}}} \\ \qquad\qquad\quad\;\,= \Delta G_{{{{\mathrm{WT}}}} \to {{{\mathrm{Mut}}}}}- \Delta G_{{{{\mathrm{WT}}}},{{{\mathrm{Drug}}}} \to {{{\mathrm{Mut}}}},{{{\mathrm{Drug}}}}}.\end{array}$$

Therefore, we obtained the following final equation:

$$\Delta \Delta G_{{{{\mathrm{coupling}}}}} = \left( {G_{{{{\mathrm{WT}}}}} + G_{{{{\mathrm{Mut}}}},{{{\mathrm{Drug}}}}}} \right) - \left( {G_{{{{\mathrm{Mut}}}}} + G_{{{{\mathrm{WT}}}},{{{\mathrm{Drug}}}}}} \right).$$

If the mutant channel and drug function are independent of each other, the coupling energy should be close to zero. If the mutant channel directly or allosterically affects drug binding, the coupling energy should be less or more than zero. The coupling energy was expressed in absolute values.

### WT and mutant KCNQ4 structure prediction and visualization

ColabFold and AlphaFold2 were used to construct homotetramer structures of the WT and mutant KCNQ4 channels^24,25^. Owing to the flexibility of the N- and C-termini and the limitation of the total length of input sequences, we only focused on the transmembrane domains of the KCNQ4 channel. This included residues 74 to 336, and the total length of the modeling residue was 1052. The ColabFold program was downloaded from the website https://github.com/sokrypton/ColabFold and run locally^25^. For protein modeling, the number of recycles was set to three, and the model type variable was AlphaFold2-multimer-v2, which is the latest available version. The top-ranked structure was used for further analysis. The HOLE program^26^ was used to calculate the ion-permeating pore region of the KCNQ4 channels. We gathered graphical pore data from HOLE and reconstructed them in ChimeraX^27^ for better visualization.

To calculate the electrostatic potential energy (Ф) identified by HOLE, we solved the linearized Poisson–Boltzmann equation in the CHARMM-GUI PBEQ solver^28–31^. The average electrostatic potential field was calculated using the CHARMM36 force field in a 127 × 127 × 79 Å^3^ space with a grid spacing of 1 Å^3^. The thickness of the membrane was 35 Å, and the dielectric constants were as follows: 2 for the protein, 2 for the membrane, 30 for the membrane headgroups, and 80 for the solvent with a monovalent ionic concentration of 150 mM.

### Fluorescence-based thallium flux assay

Plasmids containing *KCNQ4* variants or empty plasmids were transfected into CHO-K1 cells that were seeded in 96-well plates. After 48 h, the medium was replaced with 80 μL of FluxOR loading buffer (Invitrogen), and the cells were incubated for 1 h at 37 °C in the dark. The loading buffer was removed, and 100 µL of assay buffer was added to each well. To activate KCNQ4 channels, cells were pretreated with 10 μM MaxiPost for 10 min. FluxOR fluorescence (excitation/emission: 490/525 nm) was recorded 10 s prior to adding 20 µL of the stimulus buffer containing a low level of thallium ions, and the fluorescence was monitored for an additional 15 s. FluxOR fluorescence was recorded using a Zyla sCMOS camera (Andor Technology, UK) and a Nikon Eclipse Ti inverted microscope (Nikon Instruments, Tokyo, Japan) and analyzed using Metamorph analysis software (Molecular Devices). All buffers were prepared in accordance with the manufacturer’s instructions.

## Results

### *KCNQ4* variants associated with DFNA2 in patients with hearing loss and clinical assessment

**Fig. 1:**
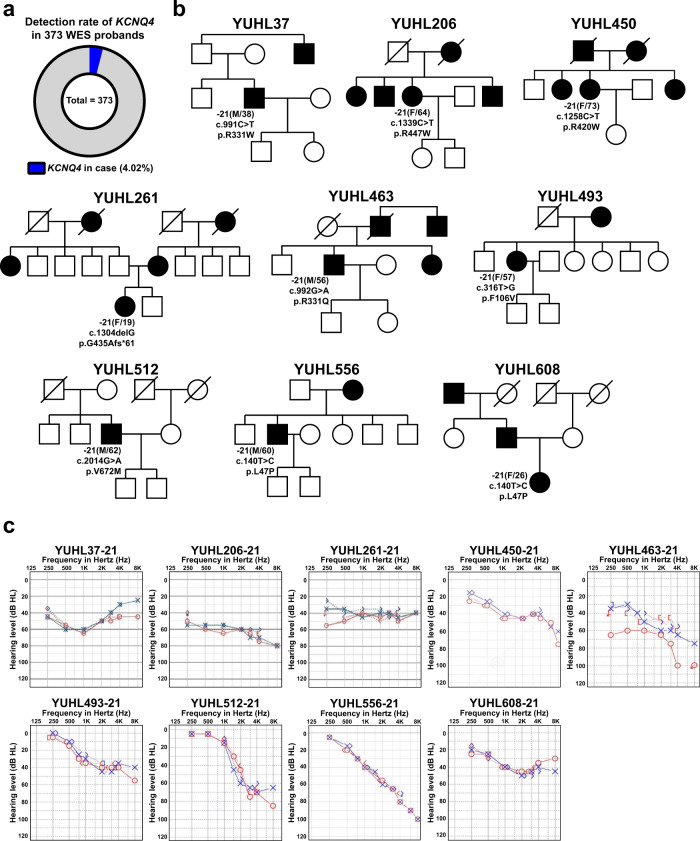
Pedigrees and pure-tone audiogram from YUHL patients. **a** Detection rate of *KCNQ4* variants in the YUHL cohort. Variants were identified in 15 unrelated families among 373 families for which WES was performed (*n* = 15/373; 4.02%). Eight of 10 *KCNQ4* variants identified in 9 families were included in this study. **b** Pedigrees of nine independent families from the Yonsei University Hearing Loss (YUHL) cohort. All of the YUHL families exhibited an autosomal dominant pattern of *KCNQ4* variants. **c** Pure-tone audiogram from the affected individuals in the YUHL families. Patients YUHL 450, 493, and 512 had high-frequency hearing loss. Patients YUHL 206 and 261 had flat-type moderate hearing loss, while Patient YUHL 463 had severe and high-frequency hearing loss.

Currently, 1,104 families are enrolled in the YUHL cohort study. To identify the genetic cause of hearing loss, we performed WES in 373 families with hearing loss after prescreening for *GJB2* and *SLC26A4*, the two most commonly mutated genes associated with hearing loss in Koreans. Among the 373 families, we detected potential causative *KCNQ4* variants in 15 families, indicating that 4.02% (15/373) of the hearing loss cases were potentially caused by KCNQ4 (Fig. 1a). From these 15 families, we identified ten rare variants, all from the well-sequenced region of KCNQ4 (Supplementary Tables 1 and 2). Excluding the previously reported variants (p.D266Y and p.V87_N89del)^16,18^, we performed a functional evaluation of seven missense variants, including p.L47P, which was previously reported as a pathogenic variant^17^, and one deletion variant in *KCNQ4* (Table 1). Among these *KCNQ4* variants, the p.G435Afs*61 variant has not been reported in public databases, including ESP, gnomAD, and the Korean Reference Genome Database (KRGDB); thus, this study is the first to report a clinical interpretation of this variant. Based on pedigrees, all families carrying *KCNQ4* variants showed an autosomal dominant pattern (Fig. 1b). Furthermore, we performed segregation analysis using Sanger sequencing in nine families and found that the variants cosegregated in patients with a progressive hearing loss phenotype (Supplementary Fig. 1). The pure-tone audiogram study revealed a high-frequency hearing loss pattern in three affected patients, YUHL450-21, YUHL493-21, and YUHL512-21, harboring the *KCNQ4* p.R420W, p.F106V, and p.V672M variants, respectively (Fig. 1c). The patients harboring KCNQ4 p.R447W and p.G435Afs*61 variants (YUHL206-21 and YUHL261-21) exhibited a flat-type moderate hearing loss pattern. Patient YUHL37-21 harboring the p.R331W variant also had moderate hearing loss (Fig. 1c), whereas Patient YUHL463-21 harboring the KCNQ4 p.R331Q variant exhibited a severe and high-frequency hearing loss pattern (Fig. 1c). The amino acid residues affected by the variants detected in the YUHL cohort were evolutionarily conserved down to *Danio rerio* (Supplementary Fig. 2).

### Effects of KCNQ4 variants identified in YUHL families on channel properties

**Fig. 2:**
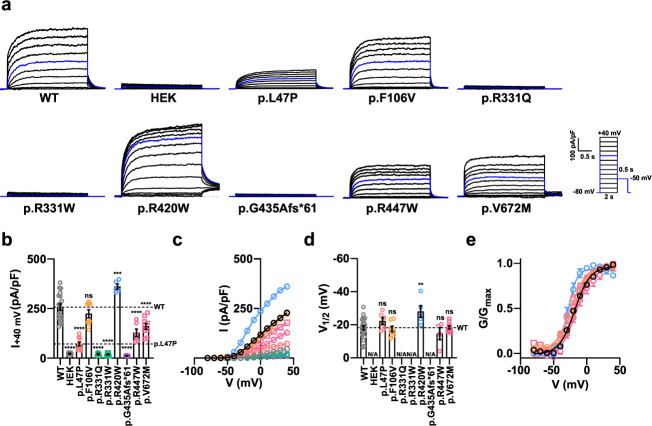
Effect of *KCNQ4* variants from the YUHL cohort on voltage-gated channel activity. **a** Whole-cell currents recorded from HEK 293 cells overexpressing WT or mutant KCNQ4 proteins identified from the YUHL cohort. From a holding potential of −80 mV, currents were evoked by depolarizing voltage steps from −80 to 40 mV in 10 mV increments. The blue line indicates the current measured at a 0 mV test pulse. **b** Current density measured at 40 mV. **c** Mean current-voltage (I-V) relationship. Trace representatives are the same as (**b**), except for the WT colored black. **d** Half-maximal voltages calculated by fitting the normalized conductance to the Boltzmann equation. The conductance was measured by calculating the magnitude of the tail currents at −50 mV after each voltage step. HEK, p.R331Q, p.R331W, and p.G435Afs*61 variants showed no measurable conductance; N/A, not available. **e** Normalized conductance-voltage (G/G_max_-V) relationship. The curves were fitted using the Boltzmann equation. Trace representatives are the same as **d**, except for the WT colored in black. Values are shown as the mean ± SEM. *P* values were calculated using one-way ANOVA, followed by Tukey’s post hoc test; ns, not significant; *p* < 0.01, **p < 0.001, ***p < 0.0001, ****.

To examine how the identified *KCNQ4* variants from the YUHL cohort affect KCNQ4-mediated K^+^ channel activity, we performed patch-clamp electrophysiology experiments to measure whole-cell currents in HEK 293 cells transiently transfected with WT or mutant KCNQ4 proteins (Fig. 2a). Cells transfected with WT KCNQ4 showed outward K^+^ currents on depolarizing voltage steps from −80 mV to 40 mV for 2 s, while negative control cells transfected with GFP alone did not (Fig. 2a). To compare the current densities of the newly identified variants, we used the p.L47P variant, which was previously reported to cause impaired KCNQ4-mediated K^+^ channel currents^17^ (Fig. 2a–c). The recorded voltage-gated K^+^ currents from the KCNQ4 variants showed varied results, similar to those exhibited by the p.R331Q variant, which is known to reduce voltage-gated K^+^ channel activity^32^. Current densities in cells expressing p.R331W and p.G435Afs*61 variants were similar to those in the negative controls. Likewise, the p.F106V variant decreased KCNQ4-mediated channel activity, but not significantly, compared with WT KCNQ4-mediated channel currents. The p.R447W and p.V672M variants also showed decreased currents compared to WT. Both variants showed larger current densities than p.L47P, which was 1/3 of that of WT KCNQ4 (71.95 ± 9.41 pA/pF). However, the current density of the p.R447W variant was 128.0 ± 19.50 pA/pF, which was half of that of WT KCNQ4 (257.7 ± 15.81 pA/pF). Thus, we assumed that the p.R447W variant might have a pathogenic effect on channel activity. The p.R420W variant showed an increased K^+^ current, unlike the other variants. We performed a surface biotinylation assay to identify the membrane expression of the KCNQ4 variants (Supplementary Fig. 3). The reduced channel activity of the p.G435Afs*61 variant correlated with the lack of plasma membrane expression. In contrast, other variants showed membrane expression profiles comparable to that of the WT protein, suggesting that these variants may directly affect channel activity.

We questioned whether the variants might also affect the intrinsic voltage sensitivity of the channel; thus, we calculated the half-maximal activation voltage (V_1/2_) by fitting the normalized voltage-conductance results to the Boltzmann equation (Fig. 2d, e). The conductance was measured by calculating the magnitude of the tail currents at −50 mV after each voltage step. The p.L47P, p.F106V, p.R447W, and p.V672M variants showed results similar to those of the WT protein, whereas the p.R420W variant showed increased voltage sensitivity (decreased V_1/2_ value) (Fig. 2d, e). p.R331Q, p.R331W, and p.G435Afs*61 variants showed no measurable conductance even at 40 mV prepulse; thus, we could not calculate V_1/2_. From the above results, we speculated that dysfunction in the p.R331Q and p.R331W channels were responsible for the lack of measurable channel currents and conductance, whereas disrupted plasma membrane expression was responsible for the dysfunctionality of the p.G435Afs*61 variant. In addition, decreased channel currents from the p.R447W variant may have deleterious effects on KCNQ4 channel function.

### *KCNQ4* variants identified from the Korean population

**Fig. 3:**
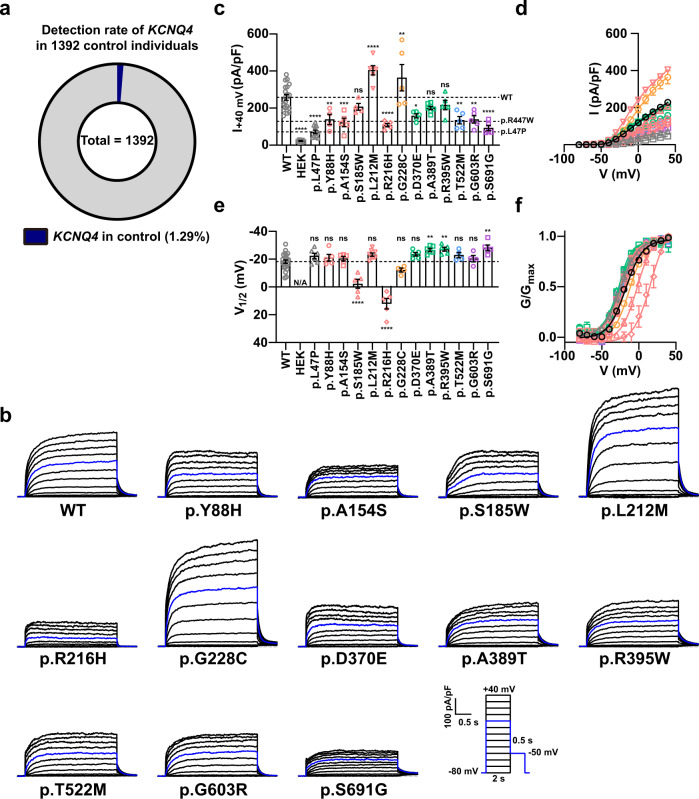
Effect of *KCNQ4* variants from the Korean population on voltage-gated channel activity. **a** Detection rate of *KCNQ4* variants in the combined Korean genomic data. Fourteen rare variants were found in 18 of 1392 control individuals with an unknown hearing loss phenotype (*n* = 18/1392; 1.29%). **b** Whole-cell currents recorded from HEK 293 cells overexpressing WT or mutant KCNQ4 proteins identified in the Korean general population. From a holding potential of −80 mV, currents were evoked by depolarizing voltage steps from −80 to 40 mV in 10 mV increments. **c** Current density measured at 40 mV. **d** Mean current-voltage (I-V) relationship. Trace representatives are the same as **c**, except for the WT colored black. The blue line indicates the current measured at a 0 mV test pulse. **e** Half-maximal voltages calculated by fitting the normalized conductance to the Boltzmann equation. The conductance was measured by calculating the magnitude of the tail currents at −50 mV after each voltage step. N/A, not available. **f** Normalized conductance-voltage (G/G_max_-V) relationship. The curves were fitted using the Boltzmann equation. Trace representatives are the same as **e**, except for the WT colored in black. Values are shown as the mean ± SEM. *p* values were calculated using one-way ANOVA, followed by Tukey’s post hoc test; ns, not significant; *p* < 0.05, *p < 0.01, **p < 0.001, ***p < 0.0001, ****.

Because of late-onset and progressive hearing loss caused by mutations in *KCNQ4*, we assumed that pathogenic *KCNQ4* variants might be overlooked in the population with an unknown hearing loss phenotype. Thus, we investigated *KCNQ4* variants in the Korean population and obtained WES data from the CODA and SNU databases, as well as additional WGS data from the KNIH and CODA databases. Using these sequencing data, we identified 14 missense variants in *KCNQ4* from the Korean population (Fig. 3a, Table 2, and Supplementary Fig. 4). Among these variants, we identified KCNQ4 p.R420W and p.R447W variants that overlapped with the YUHL family variants (Tables 1 and 2). KCNQ4 p.T522M and p.G228C variants have already been reported in ClinVar (https://www.ncbi.nlm.nih.gov/clinvar/) but were classified as having uncertain significance (VCV000504603 and VCV001032259). In addition to these two variants, six variants, including p.Y88H, p.R395W, p.R420W, p.R447W, p.G603R, and p.S691G, have been reported in gnomAD. Except for the p.R420W variant, the MAF of the other variants was very low (less than 0.0000487). In the case of the p.R420W variant, which was also identified in the YUHL cohort, the MAF was relatively high (MAF = 0.0002). The KCNQ4 p.R420W variant was also listed in the Korean Reference Genome Database (KRGDB, 1722 individuals) and exhibited a high MAF (0.00136364). In contrast, six variants, p.A154S, p.S185W, p.L212M, p.R216H, p.D370E, and p.A389T, were not reported in gnomAD, dbSNP, KRGDB, or the Exome Aggregation Consortium (ExAC). The amino acid residues affected by variants detected in the Korean population were evolutionarily conserved down to *Xenopus tropicalis* or *Danio rerio* (Supplementary Fig. 2). Most of the variants were not sufficient for classification as benign or likely benign variants according to the American College of Medical Genetics and Genomics (ACMG) guidelines; therefore, we decided to further examine the pathogenicity of these *KCNQ4* variants.

**Table 2:** *KCNQ4* variants detected in the Korean population with an unknown hearing loss phenotype.

Group^a^ (n)	Individual	Sex	Nucleotide change^b^	Amino acid change	Exon	Zygosity	Amino acid sequence conservation	dbSNP^c^	ESP^d^	gnomAD^e^	KRGDB^f^	PP2^g^	MT^h^	SIFT^i^	CADD^j^	DVD^k^
CODA WES (298)	N065	M	c.2071 A > G	p.Ser691Gly	14/14	Het	*Xenopus tropicalis*	rs200757822	ND	0.0000119	ND	Dam (0.97)	DC (0.9)	Del (0)	27.6	Unknown significance
	N056	M	c.1565 C > T	p.Thr522Met	11/14	Het	*Xenopus tropicalis*	rs748693577	ND	0.0000359	ND	Dam (0.964)	DC (0.9)	Tol (0.13)	23	Unknown significance
	CODA597, N163	M F	c.1339 C > T	p.Arg447Trp	10/14	Het	*Gallus gallus*	rs758333323	ND	0.0000335	ND	Dam (0.446)	Neu (0.98)	Del (0.02)	24.7	Unknown significance
	N079	F	c.682 G > T	p.Gly228Cys	4/14	Het	*Danio rerio*	rs367890569	0.000116	0.00001282	ND	Dam (0.977)	DC (1)	Del (0)	33	Unknown significance
CODA WGS (252)	SP000113_hg38, SP000072_hg38	F F	c.1258 C > T	p.Arg420Trp	9/14	Het	*Mus musculus*	rs576041348	ND	0.0002	0.00136364	Dam (0.548)	DC (0.9)	Del (0.02)	25.5	Unknown significance
	SP000217_hg38	F	c.1183 C > T	p.Arg395Trp	9/14	Het	*Gallus gallus*	rs373405864	0	0.0000487	0.000454545	Ben (0)	DC(1)	Del(0.03)	23.9	Unknown significance
KNIH (397)	NIH1609693850	NA	c.1165 G > A	p.Ala389Thr	9/14	Het	*Gallus gallus*	ND	ND	ND	ND	Ben (0.007)	DC (1)	Tol (0.23)	22.4	ND
SNU (445)	061293N-58N	NA	c.647 G > A	p.Arg216His	4/14	Het	*Danio rerio*	ND	ND	ND	ND	Dam (0.966)	DC (1)	Del (0)	33	ND
	130720-I433-L6-61, 195 N	NA	c.1258 C > T	p.Arg420Trp	9/14	Het	*Mus musculus*	rs576041348	ND	0.0002	0.00136364	Dam (0.548)	DC (0.9)	Del (0.02)	25.5	Unknown significance
	080223N-139N	NA	c.554 C > G	p.Ser185Trp	4/14	Het	*Danio rerio*	ND	ND	ND	ND	Dam (0.991)	DC (1)	Del (0)	32	ND
	130703-I297-L5-70	NA	c.1807G > A	p.Gly603Arg	13/14	Het	*Xenopus tropicalis*	rs755220278	ND	0.000004044	ND	Ben (0.368)	DC (0.7)	Tol (0.3)	18.96	Unknown significance
	092932N-250N	NA	c.460 G > T	p.Ala154Ser	3/14	Het	*Danio rerio*	ND	ND	ND	ND	Dam (0.999)	DC (1)	Tol (0.11)	25.8	ND
	Pat05-11252	NA	c.1110 C > A	p.Asp370Glu	8/14	Het	*Xenopus tropicalis*	ND	ND	ND	ND	Dam (0.991)	DC (0.9996)	Tol (1)	19.33	ND
	092840N-248N	NA	c.634 C > A	p.Leu212Met	4/14	Het	*Danio rerio*	ND	ND	ND	ND	Dam (0.969)	DC (1)	Del (0)	27.5	ND
	092659N-238N	NA	c.262 T > C	p.Tyr88His	1/14	Het	*Danio rerio*	rs1429420446	ND	0.00003215	ND	Dam (0.502)	DC (0.9)	Del (0.01)	29.2	Unknown significance

*Ben* benign, *Dam* probably damaging, *Del* deleterious, *Neu* neutral, *DC* disease-causing, *Tol* tolerated, *Het* heterozygous in the affected individual, *F* female, *M* male, *ND* no data or DNA not available, *Neu* neutral.

^a^Group indicates cohorts in which individuals with variants were found, comprising CODA, KNIH, and SNU.

^b^Variants are numbered according to the human cDNA reference sequence NM_004700.4 (*KCNQ4*).

^c^dbSNP database (http://www.ncbi.nlm.nih.gov/SNP).

^d^Exome Sequencing Project (https://evs.gs.washington.edu/EVS/).

^e^Population frequency of *KCNQ4* variants in gnomAD (https://gnomad.broadinstitute.org/).

^f^The Korean Reference Genome Database (http://coda.nih.go.kr/coda/KRGDB/index.jsp).

^g^PolyPhen-2 HumVar prediction score (http://genetics.bwh.harvard.edu/pph2/).

^h^MutationTaster (http://www.mutationtaster.org/).

^i^SIFT Sorting Intolerant from Tolerant (http://sift.jcvi.org/).

^j^phred-like scores (scaled C-scores) on the Combined Annotation-Dependent Depletion (http://cadd.gs.washington.edu/home/).

^k^Deafness variation database (https://deafnessvariationdatabase.org/).

### Effects of KCNQ4 variants identified in the Korean population on channel properties

We measured the K^+^ currents of *KCNQ4* variants found in the Korean population. KCNQ4 p.L47P, which showed an evident clinical phenotype and decreased current densities, was used as the “variant control”^17^. In addition, the p.R447W variant, found in both cohorts and exhibiting decreased current densities, was used as another variant control. Multiple variants, including p.Y88H, p.A154S, p.R216H, p.D370E, p.T522M, p.G603R, and p.S691G, showed decreased current densities compared to that of WT (Fig. 3b–d). Among these variants, the current densities of the p.R216H and p.S691G variants were almost as low as that of p.L47P, while those of other variants were similar to that of p.R447W. In contrast, the p.L212M and p.G228C variants showed increased current densities compared to that of the WT, while the p.S185W, p.A389T, and p.R395W variants had current densities that were not significantly different (Fig. 3b–d).

We also measured V_1/2_ of the variants in the Korean population (Fig. 3e, f). Most of the variants, including p.Y88H, p.A154S, p.L212M, p.G228C, p.D370E, p.T522M, and p.G603R, had V_1/2_ values that were not significantly different from those of WT. The p.A389T, p.R395W, and p.S691G variants showed lower V_1/2_ than WT (increased voltage sensitivity), whereas voltage sensitivities of the p.S185W and p.R216H variants were significantly impaired (Fig. 3e, f). All variants from the Korean population showed normal surface expression when examined using the surface biotinylation assay (Supplementary Fig. 3).

We identified variants overlapping between the Korean population and the YUHL cohort and examined the deleterious effects of KCNQ4 variants compared to the variant controls p.L47P and p.R447W. Overall, we confirmed that some of the *KCNQ4* variants from the Korean population indeed exhibited impaired channel activity, suggesting the possibility of overlooked hearing loss in these individuals.

### Effects of retigabine and zinc pyrithione on mutant KCNQ4 channel activity

**Fig. 4:**
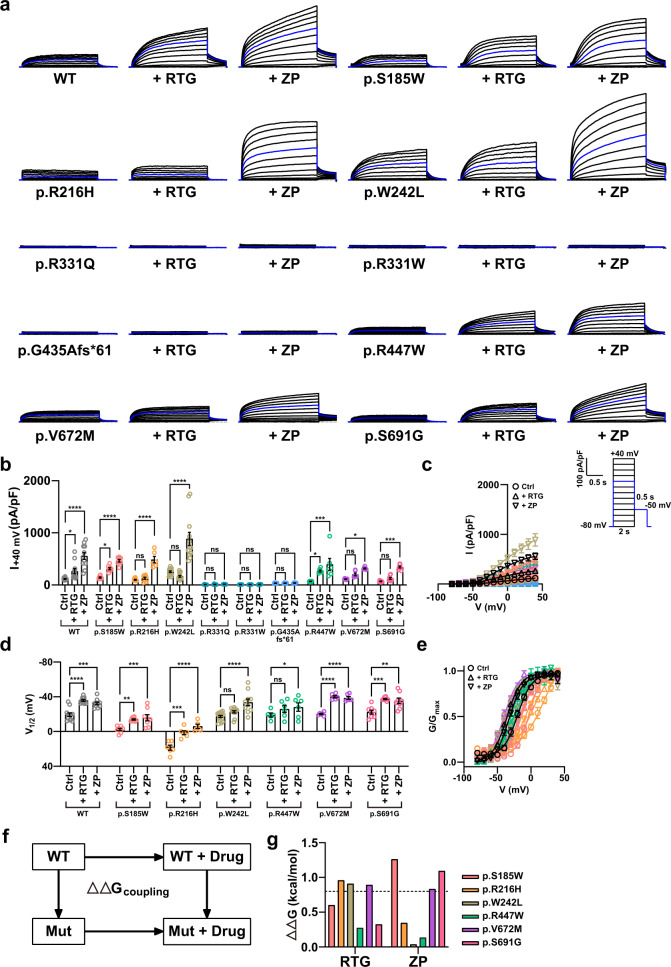
Effect of retigabine and zinc pyrithione on the WT and mutant KCNQ4 currents. **a** Whole-cell currents recorded from HEK 293 cells overexpressing WT or mutant KCNQ4. RTG and ZP represent retigabine and zinc pyrithione, respectively. Both drugs were administered at 10 μM. The blue line indicates the current measured at a 0 mV test pulse. **b** Current density measured at 40 mV. **c** Mean current-voltage (I-V) relationship. Trace representatives are the same as (**b**), except for the WT colored black. **d** Half-maximal voltages calculated by fitting the normalized conductance to the Boltzmann equation. The conductance was measured by calculating the magnitude of the tail currents at −50 mV after each voltage step. **e** Normalized conductance-voltage (G/G_max_-V) relationship. The curves were fitted using the Boltzmann equation. Trace representatives are the same as **d**, except for the WT colored in black. **f** Illustration of mutant-drug cycle analysis. $\Delta \Delta G_{coupling} = \left( {G_{WT} + G_{Mut,Drug}} \right) - \left( {G_{Mut} + G_{WT,Drug}} \right)$ was used to calculate the free energy deviation. **g** ΔΔG_coupling_ values for retigabine and zinc pyrithione with p.S185W, p.R216H, p.W242L, p.R447W, p.V672M, and p.S691G. The ΔΔG_coupling_ value of 0.8 kcal/mol was used as the cutoff for potential interactions. Values are shown as the mean ± SEM. *P* values were calculated using two-way ANOVA, followed by Tukey’s post hoc test; ns, not significant; *p* < 0.05, *p < 0.01, **p < 0.001, ***p < 0.0001, ****.

Next, we investigated whether KCNQ channel openers, such as retigabine or zinc pyrithione, could rescue the defective channel activity among KCNQ4 variants. We used the KCNQ4 p.S185W, p.R216H, p.R331Q, p.R331W, p.G435Afs*61, p.R447W, p.V672M, and p.S691G variants, which showed significantly different current densities or voltage sensitivities compared with the WT and were classified as pathogenic, to test the drug response. We also used the p.W242L variant as the drug response control, as this mutant is known to be nonresponsive to retigabine^33^. Retigabine and zinc pyrithione activated the WT KCNQ4 channel at 10 μM (Fig. 4a–e). We then tested the effect of these drugs on KCNQ4 variants. Current densities showed that retigabine and zinc pyrithione activated the mutant channels to different degrees. The p.S185W, p.R216H, p.R447W, p.V672M, and p.S691G variants responded to both drugs, while the p.W242L variant showed no difference in activity upon treatment with retigabine and only responded to zinc pyrithione (Fig. 4a–c). In addition, the p.R331Q and p.R331W variants did not respond to either retigabine or zinc pyrithione, even though the membrane expression of these variants was comparable to that of the WT KCNQ4 channel (Fig. 4a–c and Supplementary Fig. 3). p.G435Afs*61 did not respond to either drug, which was expected because this mutant did not reach the plasma membrane, possibly due to protein misfolding (Fig. 4a–c and Supplementary Fig. 3). We also examined the effect of MaxiPost using the thallium-sensitive fluorescent dye FluxOR (Supplementary Fig. 5). Through this approach, we confirmed that the channel activities of p.S185W, p.R216H, p.W242L, p.R331Q, p.R331W, and p.G435Afs*61 were not rescued by MaxiPost (Supplementary Fig. 5).

We also investigated whether retigabine and zinc pyrithione physically or allosterically interacted with KCNQ4 variants and calculated free energy differences using mutant-drug cycle analysis (Fig. 4f, g). A free energy value of 0.8 kcal/mol was used as the cutoff value to distinguish between interaction and noninteraction. This cutoff value was selected because (i) it could distinguish the retigabine-insensitive p.W242L variant between retigabine and zinc pyrithione, and (ii) the next highest energy value after 0.8 kcal/mol was 0.35 kcal/mol for p.R216H variant interaction with zinc pyrithione.

The p.R447W variant did not interact with either retigabine or zinc pyrithione, while the p.V672M variant interacted with both drugs. The KCNQ4 p.R216H and p.W242L variants interacted with retigabine but not with zinc pyrithione. The p.S185W and p.S691G variants showed the opposite effect, interacting with zinc pyrithione but not with retigabine.

Hence, except for the p.G435Afs*61 variant, the pathogenic variants in the YUHL and Korean populations could be restored upon treatment with KCNQ4 activators. Moreover, we identified potential interaction sites between the KCNQ4 variants and each drug, which could aid therapeutic drug selection.

### Effect of sodium butyrate on the surface expression of KCNQ4 p.G435Afs*61 variant

**Fig. 5:**
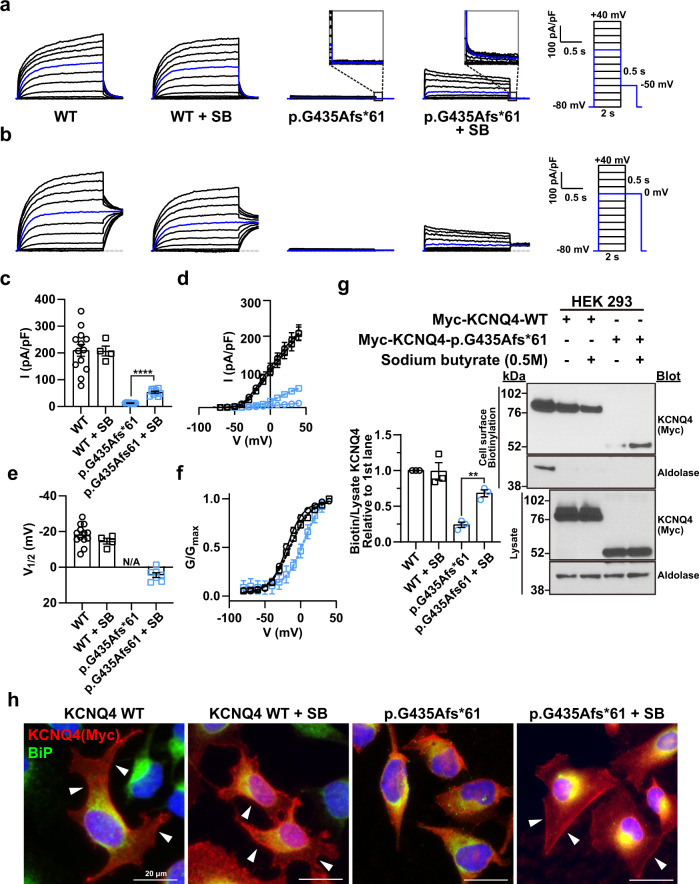
Effect of sodium butyrate on the p.G435Afs*61 variant. **a**, **b** Whole-cell currents recorded from HEK 293 cells overexpressing WT KCNQ4 or p.G435Afs*61 variant proteins treated with 0.5 M sodium butyrate (SB) for 18 h. From a holding potential of −80 mV, currents were evoked by depolarizing voltage steps from −80 to 40 mV in 10 mV increments. The tail currents were set to −50 mV in (**a**) and 0 mV in (**b**) after each voltage step. The blue line indicates the current measured at a 0 mV test pulse. **c** Current density measured at 40 mV. **d** Mean current-voltage (I-V) relationship. Trace representatives are the same as in (**c**). **e** Half-maximal voltages calculated by fitting the normalized conductance to the Boltzmann equation. **f** Normalized conductance-voltage (G/G_max_-V) relationship. The curves were fitted using the Boltzmann equation. Trace representatives are the same as (**e**). **g** Immunoblotting with surface biotinylation assay of WT and p.G435Afs*61 variant treated with 0.5 M sodium butyrate (SB) for 18 h. **h** Immunofluorescence of WT and p.G435Afs*61 variant treated with 0.5 M SB in HeLa cells. Arrows indicate the membrane expression of KCNQ4 WT and p.G435Afs*61 proteins. Values are shown as the mean ± SEM. *p* values were calculated using one-way ANOVA, followed by Tukey’s post hoc test; *p* < 0.01, **p < 0.0001, ****.

Next, we focused on the p.G435Afs*61 variant, which was nonresponsive to KCNQ activators owing to its lack of surface expression. Immunofluorescence analysis revealed that the p.G435Afs*61 variant was not detected at the cell surface and was highly colocalized with ER markers, whereas WT and other variants were detected at the cell surface (Supplementary Fig. 6). We hypothesized that protein misfolding of the p.G435Afs*61 variant disrupted ER-to-Golgi-dependent membrane trafficking. To identify whether the p.G435Afs*61 variant was transported to the cell surface via an unconventional trafficking route, we used the dominant-negative Arf1 p.Q71L mutant in which the conventional ER-to-Golgi pathway was blocked. We observed that the p.G435Afs*61 variant was not transported to the cell surface, whereas the WT reached the cell surface (Supplementary Fig. 7), suggesting that an unconventional trafficking route does not exist for p.G435Afs*61. Sodium butyrate, which is known to be effective in trafficking the misfolded CFTR ΔF508 variant to the plasma membrane^34^, was selected as the drug candidate. Surprisingly, sodium butyrate partially rescued the p.G435Afs*61 variant-mediated K^+^ current, whereas no difference in the current density or V_1/2_ was observed in WT KCNQ4 upon treatment (Fig. 5a–f). We changed the holding potential to 0 mV to obtain larger tail currents for better normalization and calculated the V_1/2_ of the p.G435Afs*61 variant upon sodium butyrate treatment (Fig. 5b). V_1/2_ of the p.G435Afs*61 variant was larger than that of the WT or WT treated with sodium butyrate, implying that it is less sensitive to voltage (Fig. 5e, f). Consistent with the patch-clamp data, the surface biotinylation assay revealed that the cell surface expression of the p.G435Afs*61 variant increased upon sodium butyrate treatment (Fig. 5g). Moreover, immunofluorescence analysis indicated that the p.G435Afs*61 protein showed a membrane pattern of localization in the presence of sodium butyrate (Fig. 5h).

**Fig. 6:**
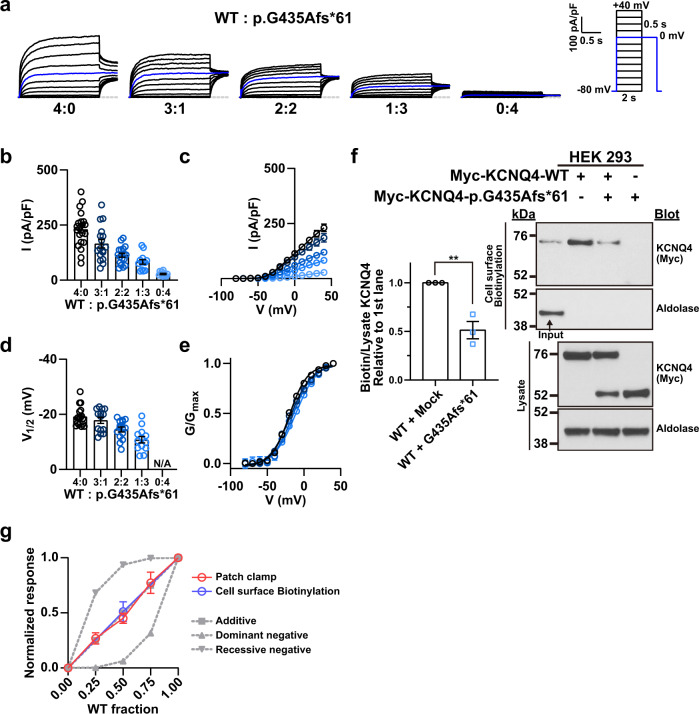
Cotransfection of the p.G435Afs*61 variant with WT KCNQ4. **a** Whole-cell currents recorded from HEK 293 cells overexpressing WT KCNQ4 with the p.G435Afs*61 variant proteins. From a holding potential of −80 mV, currents were evoked by depolarizing voltage steps from −80 to 40 mV in 10 mV increments. The blue line indicates the current measured at a 0 mV test pulse. **b** Current density measured at 40 mV. **c** Mean current-voltage (I-V) relationship. Trace representatives are the same as (**b**). **d** Half-maximal voltages calculated by fitting the normalized conductance to the Boltzmann equation. The conductance was measured by calculating the magnitude of the tail current at 0 mV after each voltage step. **e** Normalized conductance-voltage (G/G_max_-V) relationship. The curves were fitted using the Boltzmann equation. Trace representatives are the same as (**d**). **f** Immunoblotting with surface biotinylation assay of cotransfected WT and p.G435Afs*61 variant. **g** WT fraction-normalized response relationship. Additive, dominant-negative, and recessive negative traces are shown in gray; the patch clamp trace of p.G435Afs*61 with WT is shown in red; and the biotin trace is shown in blue. The normalized responses were calculated using (**b** and **f**). Values are shown as the mean ± SEM.

**Fig. 7:**
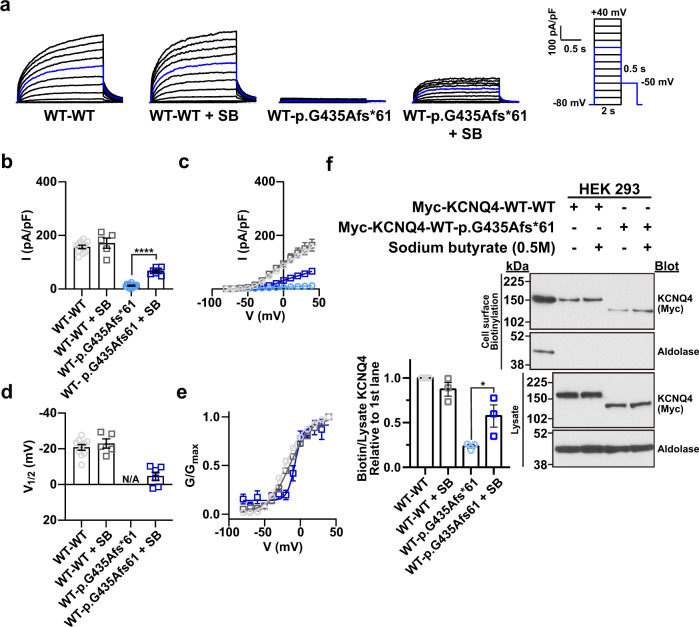
Effect of sodium butyrate on p.G435Afs*61 tandem concatemer. **a** Whole-cell currents recorded from HEK 293 cells overexpressing the WT-WT KCNQ4 or WT-p.G435Afs*61 variant tandem concatemers treated with 0.5 M sodium butyrate (SB) for 18 h. From a holding potential of −80 mV, currents were evoked by depolarizing voltage steps from −80 to 40 mV in 10 mV increments. The blue line indicates the current measured at a 0 mV test pulse. **b** Current density measured at 40 mV. **c** Mean current-voltage (I-V) relationship. Trace representatives are the same as (**b**). **d** Half-maximal voltages calculated by fitting the normalized conductance to the Boltzmann equation. The conductance was measured by calculating the magnitude of the tail currents at 0 mV after each voltage step. **e** Normalized conductance-voltage (G/G_max_-V) relationship. The curves were fitted using the Boltzmann equation. Trace representatives are the same as (**d**). **f** Immunoblotting with surface biotinylation assays of WT-WT and WT-p.G435Afs*61 tandem concatemers treated with 0.5 M SB for 18 h. Values are shown as the mean ± SEM. *p* values were calculated using one-way ANOVA and Student’s *t* test, followed by Tukey’s post hoc test; *p* < 0.05, *p < 0.0001, ****.

Next, we investigated whether expressing the WT KCNQ4 channel with the p.G435fs*61 variant rescued channel activity. Cotransfection of the WT and p.G435fs*61 variant at 4:0, 3:1, 2:2, 1:3, and 0:4 ratios resulted in a stepwise decrease in the current density and V_1/2_ (Fig. 6a–e). Consistently, the membrane expression of WT KCNQ4 was reduced by 50% when cotransfected with the p.G435Afs*61 variant (Fig. 6f). The relationship between the normalized current and the fraction of the WT channel followed the additive model rather than the dominant-negative model (Fig. 6g). We then generated tandem KCNQ4 concatemers that enabled us to obtain currents from a fixed ratio of 1:1 and forced heteromerization (Fig. 7). The WT-WT concatemer showed current density and V_1/2_ values comparable to those of the WT. Surprisingly, the WT-p.G435Afs*61 concatemer showed no sign of channel activity, exhibiting a dominant-negative effect (Fig. 7a–c). When treated with sodium butyrate, the current density and V_1/2_ of the WT-p.G435Afs*61 concatemer was partially rescued (Fig. 7a–c). The current density was similar to that of the p.G435Afs*61 variant, while the V_1/2_ was higher (lower voltage sensitivity) (Fig. 7a–f). The surface biotinylation assay revealed that cell surface expression of the WT-p.G435Afs*61 concatemer increased upon sodium butyrate treatment (Fig. 7f).

Overall, our electrophysiology and surface biotinylation experiments revealed unique properties of the p.G435Afs*61 variant, namely, an additive effect when cotransfected with the WT, a dominant-negative effect in the tandem concatemer, and partially rescued membrane trafficking upon treatment with sodium butyrate.

### Structure predictions of KCNQ4 variants using AlphaFold2

**Fig. 8:**
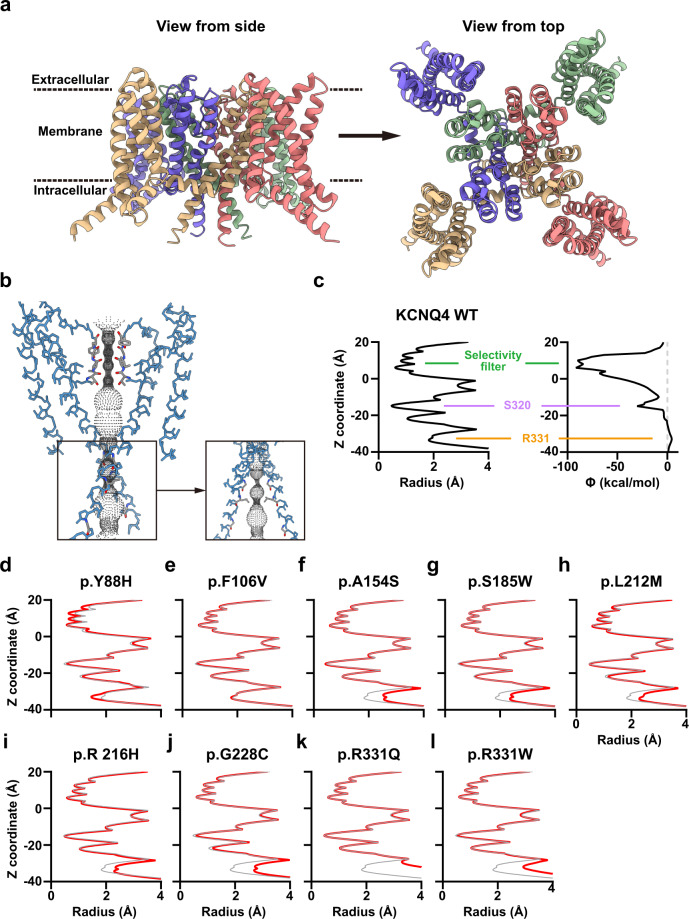
Structure of WT and mutant KCNQ4 proteins predicted using AlphaFold2. **a** Structure of the WT KCNQ4 channel predicted using AlphaFold2. **b** Pore configuration of the WT KCNQ4 channel. The pore radius and electrostatic potential energy (Ф) were calculated using the HOLE and linearized Poisson–Boltzmann equations, respectively. Residues that are important for channel function are described. **c–l** Pore radius of KCNQ4 variants. The pore radius of each variant is colored in red and that of the WT is in gray.

Next, we constructed 3D structures of KCNQ4 variants using AlphaFold2 and ColabFold^24,25^. Residues between transmembrane domains 1–6 were included (residues 74–336). We obtained a successful pore structure of WT KCNQ4, and the ion conduction pathway identified using HOLE showed similar results to the newly discovered cryo-EM structures^35,36^ (Fig. 8a). When we calculated the electrostatic potential energy of the pore by solving the linearized Poisson–Boltzmann equation^28–31^, we observed a highly negative potential at the selectivity filter, implying conductance of positively charged K^+^ ions (Fig. 8b). A comparison of the pore radius with the electrostatic potential energy revealed important residues for ion conduction: residues at the selectivity filter, S320 and R331 (Fig. 8c).

We then constructed the structures of the KCNQ4 variants located between residues 74 and 336. The p.Y88H variant, which had a smaller current density than the WT, showed collapsed pores in the selectivity filter (Fig. 8d). Surprisingly, the p.F106V variant, which showed no difference in current density or V_1/2_ in our patch-clamp data, showed the same pore conformation as the WT (Fig. 8e). The p.A154S, p.S185W, and p.R216H variants with smaller current density and/or impaired voltage sensitivity showed different pore properties near the R331 region (Fig. 8f, g, i). The p.L212M and p.G228C variants, which had higher current densities than the WT, also showed different pore structures near the R331 region (Fig. 8h, j). As expected, the pores of p.R331Q and p.R331W variants were drastically different around the R331 region (Fig. 8k, l). Overall, our structural prediction of the KCNQ4 variants confirmed that the patch-clamp data and predicted pore configuration results were consistent.

## Discussion

Herein, we studied novel *KCNQ4* mutations in the YUHL cohort and examined their ion channel functions using electrophysiology and molecular biology. Among the YUHL cohort patients, we identified novel *KCNQ4* variants, such as p.R331Q, p.R331W, p.R420W, p.G435Afs*61, p.R447W, and p.V672M. Mutations in *KCNQ4* cause autosomal dominant nonsyndromic sensorineural hearing loss (DFNA2, OMIM 600101), which is progressive across all frequencies, and affected individuals exhibit late onset. Thus, we hypothesized that overlooked variants might exist in the general population with an unknown hearing loss phenotype. We analyzed the WES and WGS data obtained from the Korean population cohort and found 14 missense variants.

Among these 14 variants, we identified two variants, p.R420W and p.R447W, that overlapped with the YUHL cohort (Tables 1 and 2). Two individuals from the CODA WGS and SNU databases carried the p.R420W variant. Moreover, the p.R420W variant not only exhibited a higher MAF value in both gnomAD (MAF = 0.0002) and KRGDB (MAF = 0.00136364) than the other variants but also showed increased current densities compared to WT. Thus, we concluded that the p.R420W variant was unlikely to be a pathogenic variant.

Unlike the p.R420W variant, the p.R447W variant exhibited a low gnomAD MAF value (0.0000335) and significantly decreased KCNQ4-mediated current density. Although the p.R447W variant did not affect KCNQ4-mediated currents as much as the p.L47P variant, the current density decreased to half of that of the WT. In a previous study, the electrophysiology data showed the pore region variants p.T278A, p.S273A, p.L281M, and p.L295P, as well as p.R433W, produced currents of ~40–53% of WT KCNQ4^37^. Thus, we classified the p.R447W variant as a pathogenic variant and used it as a “variant control” that was milder than p.L47P. Interestingly, this variant was also found in the Korean population with an unknown hearing phenotype. An individual with p.R447W is likely to develop or have hearing loss based on the fact that individuals with the p.R447W variant in the YUHL cohort had postlingual and progressive hearing loss.

Based on p.L47P-mediated currents, the variants p.S185W, p.R216H, and p.S691G were classified as pathogenic candidates, and these responded well to the KCNQ activators retigabine or zinc pyrithione, providing therapeutic possibilities for patients harboring these mutations. Similarly, based on the p.R447W-mediated currents, p.Y88H, p.A154S, p.D370E, p.T522M, p.G603R, and p.R447W could also be classified as pathogenic variants; however, further investigation into their potential as therapeutic targets is needed. Furthermore, additional research is required to determine how the p.L47P variant impairs channel function despite the mutation being located in the N-terminus.

Among the nine potentially pathogenic candidates found in the Korean population, five variants are available in the gnomAD database with MAFs (q), and the frequencies of individuals who carry these variants in a homozygous or heterozygous state (2pq + q^2^) can be calculated. Assuming that these variants were in Hardy-Weinberg equilibrium (p^2^ + 2pq + q^2^ = 1, p + q = 1), the frequencies of heterozygous or homozygous individuals with p.Y88H, p.R447W, p.T522M, p.G603R, and p.S691G were estimated to be 0.00006429, 0.00006699, 0.00007179, 0.00000808, and 0.00002379, respectively. The sum of frequencies was 0.0002349, which is ~11.75% of the prevalence of hearing loss (1/500 = 0.002)^1^. Therefore, *KCNQ4* variants may contribute more to hearing loss than expected. However, many individuals with *KCNQ4* variants probably do not recognize that they actually harbor *KCNQ4* variants and may think that their hearing deficit is age-related or noise-induced, as hearing loss resulting from *KCNQ4* variants is late-onset and progressive.

Among the variants identified in the case group, the p.G435Afs*61 variant was unique in its pathogenicity because it caused impaired membrane trafficking. In colocalization studies with ER markers using immunofluorescence, we found that the p.G435Afs*61 variant accumulated in the ER. When sodium butyrate, a chemical chaperone, was used to treat HEK 293 cells overexpressing the KCNQ4 p.G435Afs*61 variant, channel activity was partially rescued. The p.G435Afs*61 variant has a normal pore region, but the C-terminus is truncated. According to recently identified cryo-EM structures, calmodulin binds to the C-terminus of KCNQ4. It has been shown that calmodulin is involved in the inactivation of the KCNQ4 channel when the cytosolic Ca^2+^ concentration is high^38^. We speculate that calmodulin may play a larger role than a Ca^2+^ sensor because the p.G435Afs*61 variant did not respond to the KCNQ activators retigabine or zinc pyrithione even when membrane trafficking was potentiated by sodium butyrate. Further studies are required to uncover the multifaceted roles played by calmodulin in KCNQ4 channel function.

Many KCNQ activators have been discovered, including retigabine^39,40^, zinc pyrithione^41^, MaxiPost^39^, and ML213^42^. While recent cryo-EM structures have provided insights into retigabine^35^ and ML213^36^, the binding sites for the other drugs remain to be discovered. Through mutant-drug cycle analysis, we calculated the free energy difference between mutant and WT KCNQ upon drug binding. This method allowed us to observe different binding mechanisms for retigabine and zinc pyrithione; R216, W242, and V672 were crucial for binding with retigabine, while S185, V672, and S691 were important for activation by zinc pyrithione. While W242 was initially known as the direct binding site of retigabine^33^, we propose that R216 could also allosterically modulate the effect of retigabine because it is one of four voltage-sensing residues^35^. One of the major obstacles in developing KCNQ4 activators is that the activators mentioned earlier are nonspecific for all KCNQ channels. Thus, identifying the drug-binding site for KCNQ4 and other KCNQ channels is important because it could pave the way for rational drug design based on structural information.

The prediction of novel protein structures using AlphaFold has been successful in recent years, and the recently published AlphaFold2 includes features for homo- or heteromultimer predictions^24^. We initially used AlphaFold2 with ColabFold^25^ to construct S1–S6 of WT KCNQ4, which gave us results similar to the recently discovered KCNQ4 structures. As a single residue change can affect the stability or configuration of the entire protein, we modeled each variant by changing the initial input residues in AlphaFold2. Surprisingly, dysfunctional variants verified from the patch-clamp data showed impaired pores around the selectivity filter or R331 regions. While R331 has only been highlighted as a linopirdine-binding site^35^, we propose that R331 may play a more significant role because (i) p.R331Q and p.R331W variants showed zero conductance and no response to the applied voltage, and (ii) sudden narrowing of the pore could be observed at R331.

We also highlighted the possibility of utilizing AlphaFold2 to predict the pathogenicity of *KCNQ4* variants based on the pore region. As mentioned previously, the heterogeneity of *KCNQ4* mutations in hearing loss greatly impairs the chance of early diagnosis, and our findings in the Korean population, such as the p.S185W and p.R216H variants, support this statement. The main pathophysiology of hearing loss resulting from KCNQ4 mutation is reduced K^+^ recycling and continuous depolarization of the OHCs, ultimately leading to OHC death. Thus, we speculate that starting treatment at the time of diagnosis could be too late because the OHC is already lost, so early diagnosis with prophylactic treatment is crucial. In this context, our finding regarding the pathogenicity of the KCNQ4 variant using AlphaFold2 highlights the medical utilization of *de novo* protein structure prediction. Further studies are warranted to explore this possibility.

## Supplementary information

Supplementary Information
