# Supplementary material for: Overlooked KCNQ4 variants augment the risk of hearing loss
Source: Exp Mol Med. 2023 Apr 3;55(4):844–59. doi: 10.1038/s12276-023-00976-4 (PMC10167218; doi:10.1038/s12276-023-00976-4)
Supplement: Supplementary file 1 — Supplementary Information [file 12276_2023_976_MOESM1_ESM.pdf]

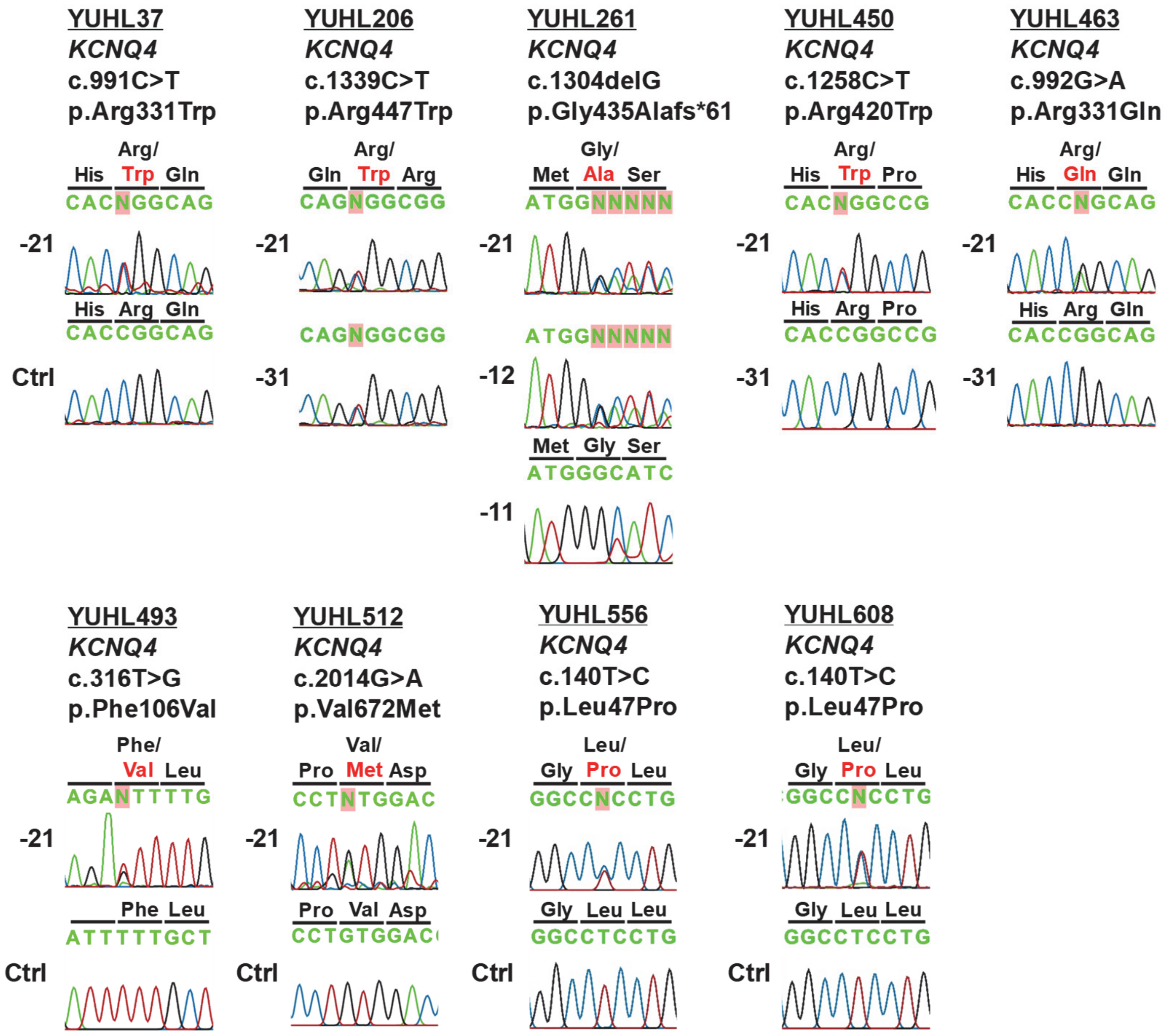

Supplementary Fig. 1. Segregation of variants in KCNQ4 in YUHL family.
Sanger sequencing traces of KCNQ4 variants are shown for individuals from YUHL families.

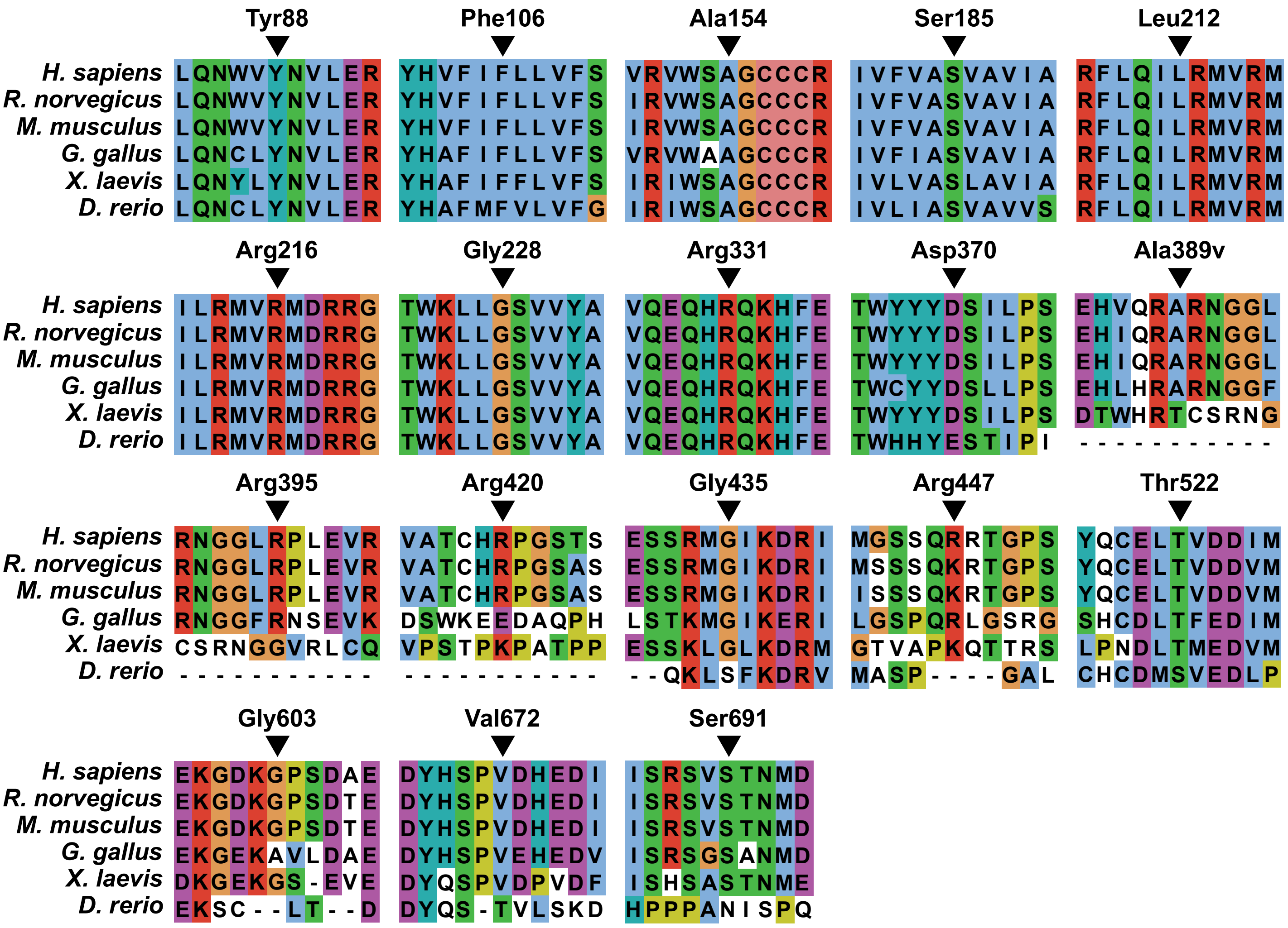

Supplementary Fig. 2. Evolutionary conservation of amino acid of 19 KCNQ4 variants.

Evolutionary conservation of amino acid residues in KCNQ4 were altered by the variants detected in the YUHL cohort and Korean general population.

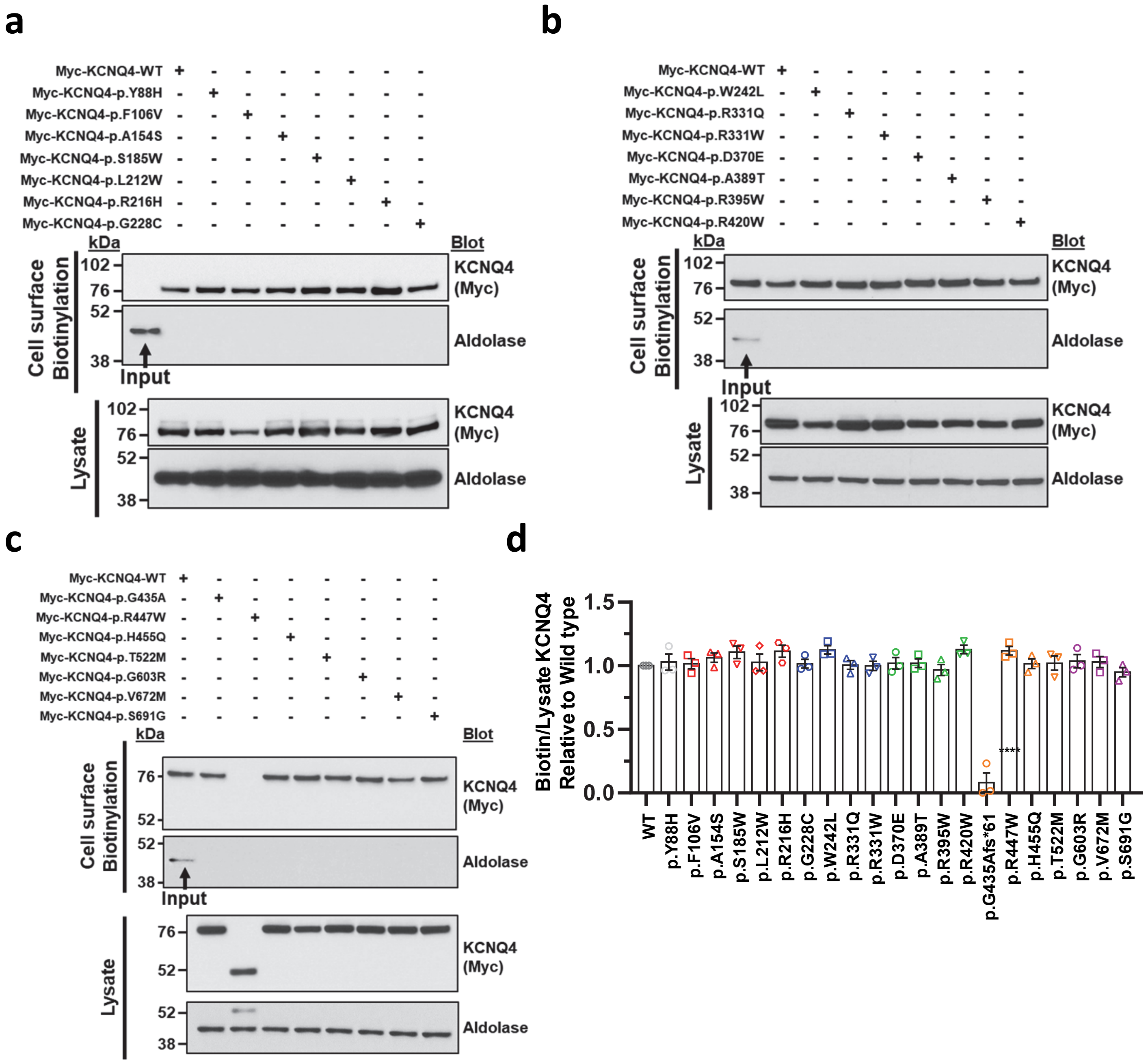

Supplementary Fig. 3. Effect of KCNQ4 variant on the membrane expression. Cell surface biotinylation of HEK293T cell overexpressing KCNQ4 variants. Membrane proteins were labeled with biotin, isolated with avidin beads. Western blot result indicated that, membrane expression of mutant KCNQ4 proteins was similar with wild-type KCNQ4 protein, except mutant KCNQ4 p.G435Afs*61 truncated protein. Truncated KCNQ4 protein showed a trafficking dysfunction. Statistical analysis was performed using one-way ANOVA with Bonferroni's multiple comparison. Data represent the mean ± SEM. ****P <0.001 compared to WT.

a. p.Ala154Ser

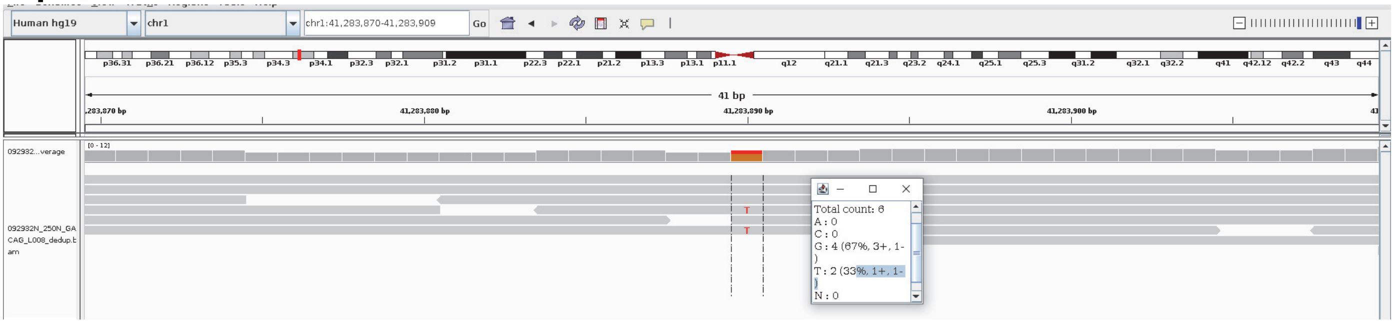

b. p.Ser185Trp

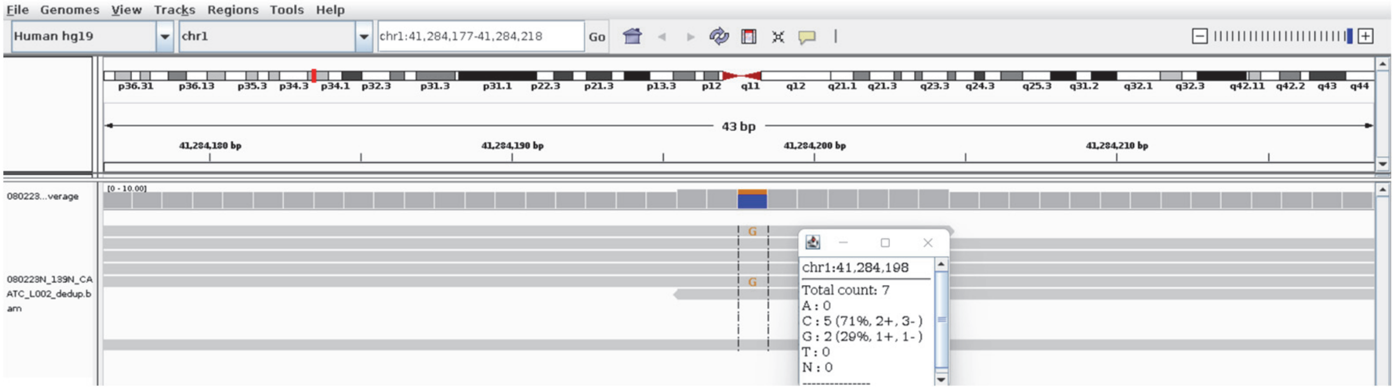

c. p.Leu212Met

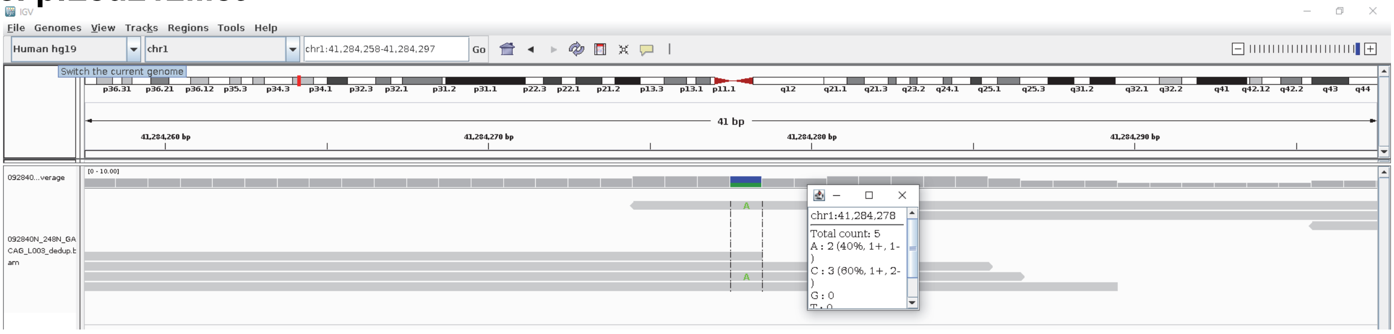

d. p.Arg216His

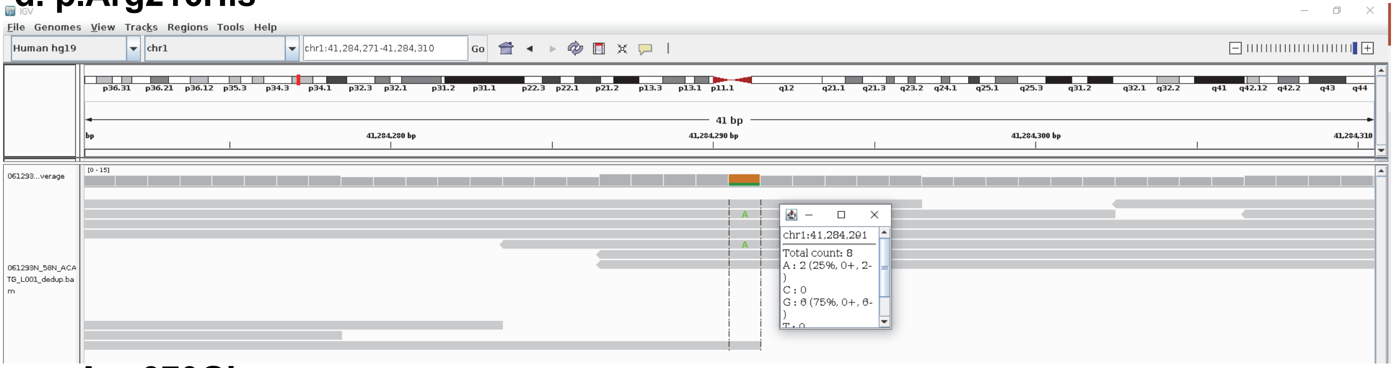

e. p.Asp370Glu

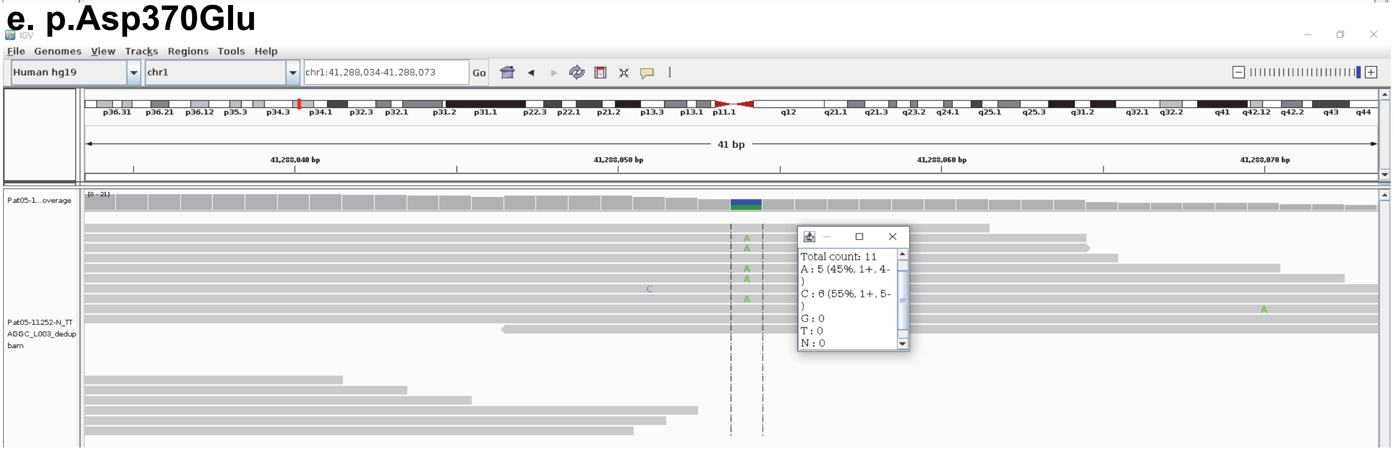

Supplementary Fig. 4 . IGV visualization of five novel *KCNQ4* variants identified in control WGS individuals.

The five missense variants are p.Ala154Ser, p.Ser185Trp, p.Leu212Met, p.Arg216His and p. Asp370Glu.

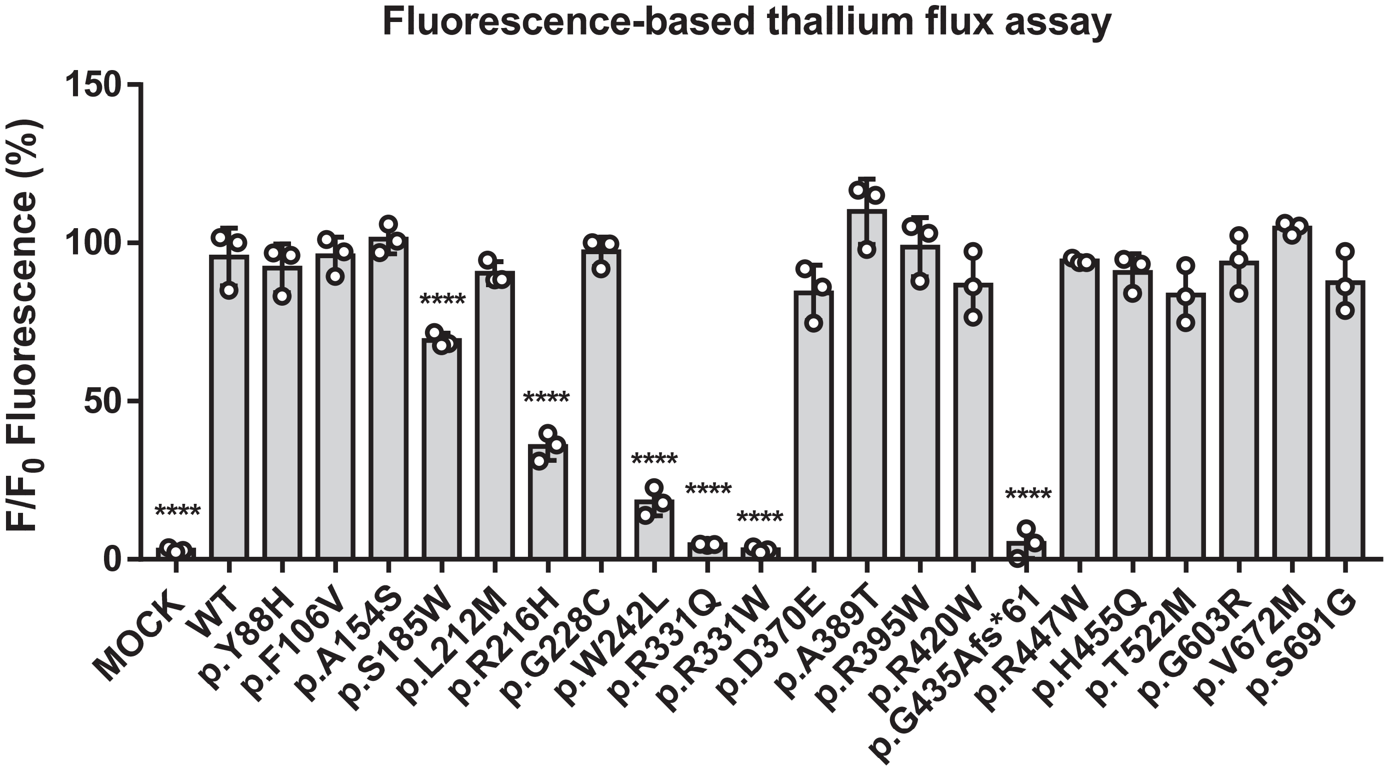

Supplementary Fig. 5. Effect of Maxipost on current produced by KCNQ4 mutants. Effect of Maxipost (10μM) on thallium influx in CHO-K1 cells overexpressing WT and mutant KCNQ4. Statistical analysis was performed using one-way ANOVA with Bonferroni's multiple comparison. Data represent the mean ± SEM. ****P <0.001 compared to WT

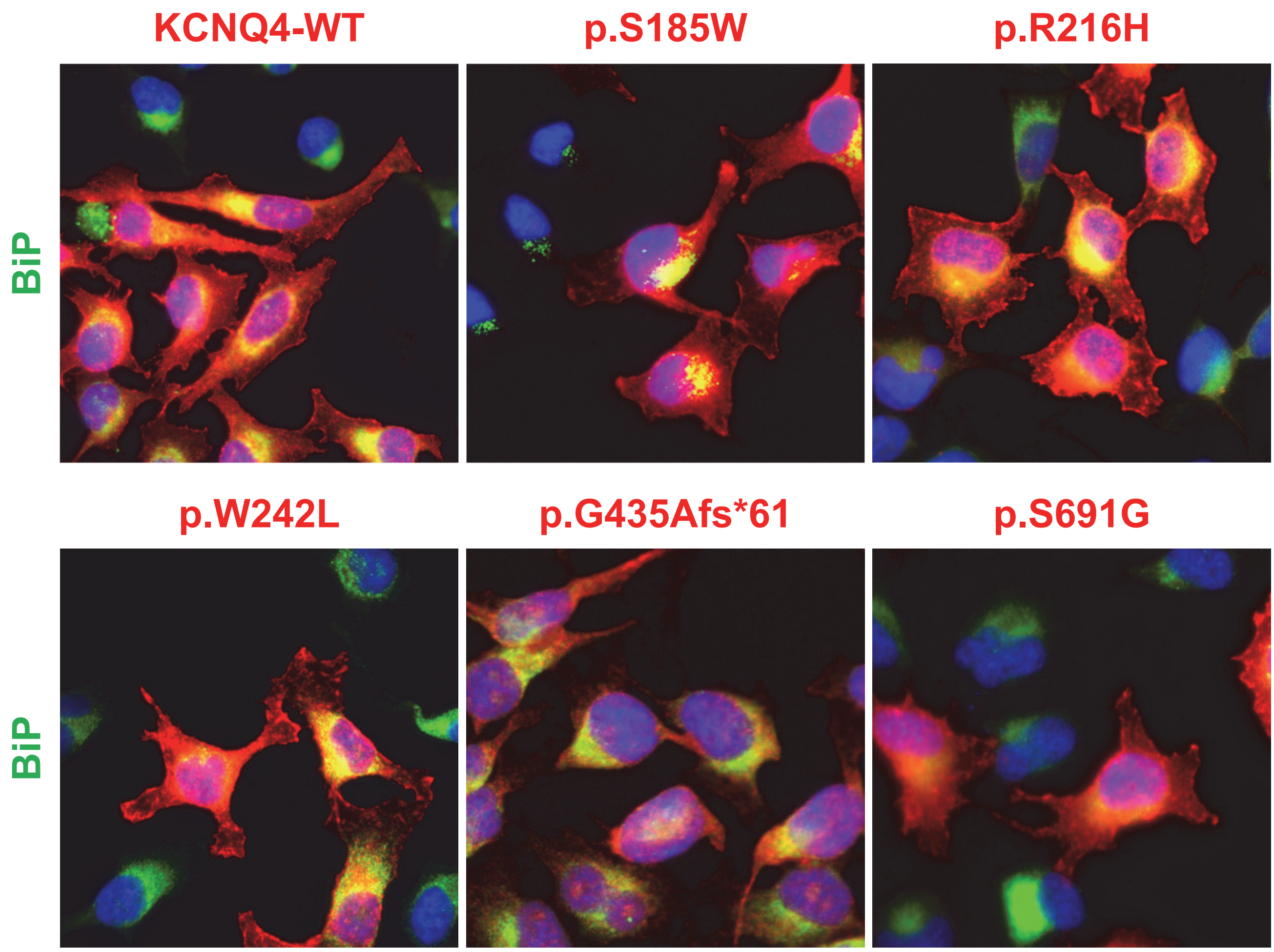

Supplementary Fig. 6. Immnofluorescence of WT and mutant KCNQ4 proteins in HeLa cells.

Cells were immunostained with anti-Myc (red) and anti-BiP (Green) antibodies, and Nuclei were stained with DAPI (blue). Protein localization of Wild-type and mutant KCNQ4 p.S185W, p.R216H, p.W242 and p.S691G proteins showed membrane pattern, mutant KCNQ4 p.G435Afs*61 protein was highly colocalized with BiP and did not show membrane pattern.

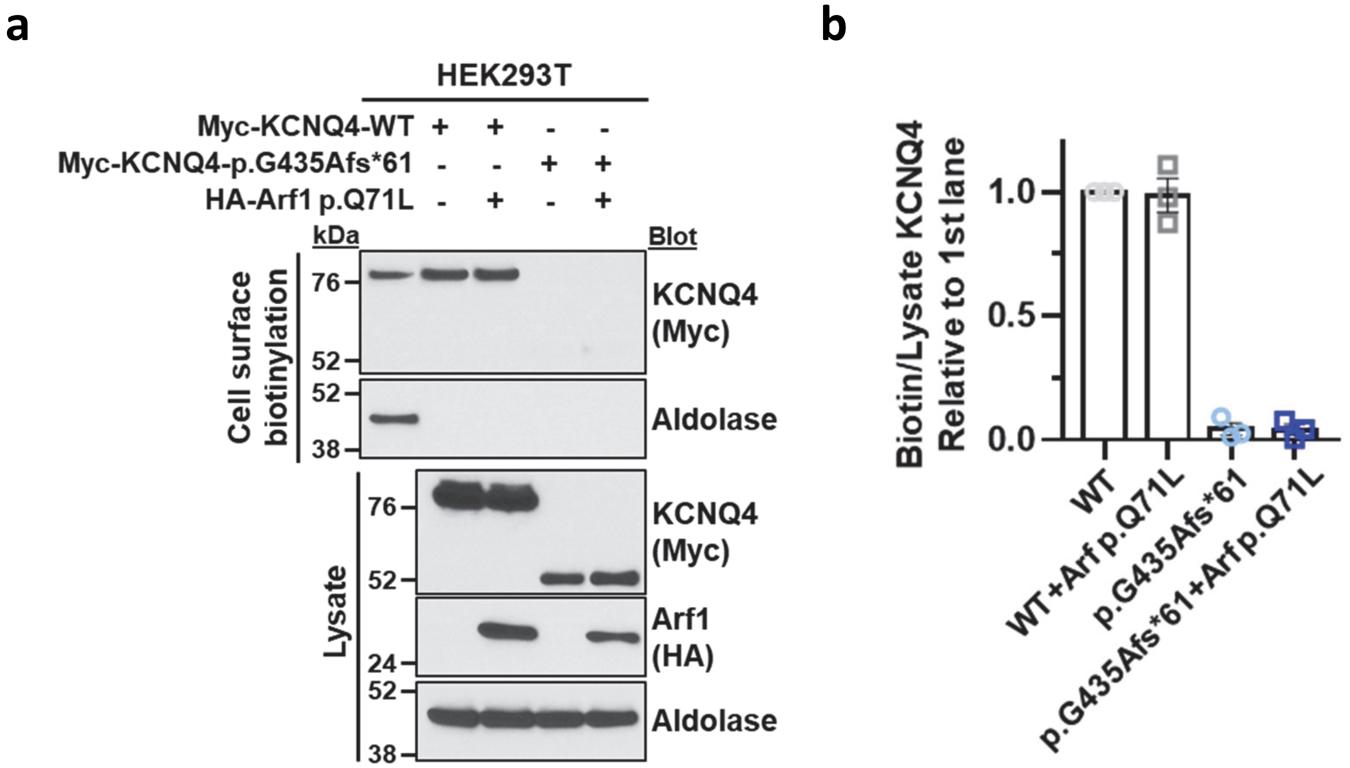

Supplementary Fig. 7. Effect of unconventional trafficking on membrane expression of KCNQ4 p.G435Afs*61.

Cell surface biotinylation of HEK293T cell overexpressing KCNQ4 WT and p.G435Afs*61 variant. Blockade of conventional ER-to-Golgi traffic by Arf1-Q71L overexpression does not induce the cell surface expression of p.G435Afs*61 variant. Data represent the mean ± SEM.

Supplementary Table 1. Mean depth of WES individuals in exonic region of *KCNQ4*. Sequencing depth was calculated using mosdepth. Exonic region of *KCNQ4* was referred to the coding sequence region of *KCNQ4* in NCBI reference sequence database (refseq).

Group (WES)	Mean Depth (95% CI)
Case	59.9 (57.8-62.0)
Control (SNU, CODA-WES)	75.5 (70.6-80.4)

Supplementary Table 2. Sequencing depth of *KCNQ4* in individuals with rare variants. The percent of bases with coverage beyond 15 was calculated using DepthOfCoverage in GATK4. Among the samples identified with rare variants in *KCNQ4*, all of cases (underlined) and 5 of 13 samples in control group showed 90% of coverage in exonic region of *KCNQ4*.

% of coverage above 15	Sample ID
**95%**	YUHL206-21, YUHL463-21, YUHL450-21, YUHL556-21, YUHL261-21, YUHL37-21, YUHL493-21, YUHL512-21, YUHL608-21
**90%**	CODA597, N056, N065, N079, N163
**50%**	Pat05-11252, 130703-I297-L5-70, 130720-I433-L6-61, Pat05-11252
**20%**	061293N-58N, 080223N-139N, 092659N-238N, 092840N-248N, 092932N-250N
